# The genome of a wild *Medicago* species provides insights into the tolerant mechanisms of legume forage to environmental stress

**DOI:** 10.1186/s12915-021-01033-0

**Published:** 2021-05-06

**Authors:** Tianzuo Wang, Lifei Ren, Caihong Li, Di Zhang, Xiuxiu Zhang, Gang Zhou, Dan Gao, Rujin Chen, Yuhui Chen, Zhaolan Wang, Fengling Shi, Andrew D. Farmer, Yansu Li, Mengyan Zhou, Nevin D. Young, Wen-Hao Zhang

**Affiliations:** 1 State Key Laboratory of Vegetation and Environmental Change, Institute of Botany, The Chinese Academy of Sciences, Beijing, China; 2 Novogene Bioinformatics Institute, Beijing, China; 3 School of Life Sciences, Lanzhou University, Lanzhou, China; 4 Institute of Grassland Research, Chinese Academy of Agricultural Sciences, Huhehot, China; 5 College of Ecology and Environmental Science, Inner Mongolia Agricultural University, Huhehot, China; 6 National Centre for Genome Resources, Santa Fe, New Mexico USA; 7 Institute of Vegetables and Flowers, Chinese Academy of Agricultural Sciences, Beijing, China; 8 Departments of Plant Pathology and Plant Biology, University of Minnesota, Minnesota, USA; 9 Inner Mongolia Research Centre for Prataculture, The Chinese Academy of Sciences, Beijing, China

**Keywords:** Wild genetic resource, Genome, Evolution, Domestication, Comparative genomics, Transcriptome, Single-nucleotide polymorphism, Drought tolerance, *Medicago ruthenica*

## Abstract

**Background:**

*Medicago ruthenica*, a wild and perennial legume forage widely distributed in semi-arid grasslands, is distinguished by its outstanding tolerance to environmental stress. It is a close relative of commonly cultivated forage of alfalfa (*Medicago sativa*). The high tolerance of *M. ruthenica* to environmental stress makes this species a valuable genetic resource for understanding and improving traits associated with tolerance to harsh environments.

**Results:**

We sequenced and assembled genome of *M. ruthenica* using an integrated approach, including PacBio, Illumina, 10×Genomics, and Hi-C. The assembled genome was 904.13 Mb with scaffold N50 of 99.39 Mb, and 50,162 protein-coding genes were annotated. Comparative genomics and transcriptomic analyses were used to elucidate mechanisms underlying its tolerance to environmental stress. The expanded *FHY3/FAR1* family was identified to be involved in tolerance of *M. ruthenica* to drought stress. Many genes involved in tolerance to abiotic stress were retained in *M. ruthenica* compared to other cultivated *Medicago* species. Hundreds of candidate genes associated with drought tolerance were identified by analyzing variations in single nucleotide polymorphism using accessions of *M. ruthenica* with varying tolerance to drought. Transcriptomic data demonstrated the involvements of genes related to transcriptional regulation, stress response, and metabolic regulation in tolerance of *M. ruthenica.*

**Conclusions:**

We present a high-quality genome assembly and identification of drought-related genes in the wild species of *M. ruthenica*, providing a valuable resource for genomic studies on perennial legume forages.

**Supplementary Information:**

The online version contains supplementary material available at 10.1186/s12915-021-01033-0.

## Background

*Medicago ruthenica* (L.) Trautv., an allogamous diploid (2*n*=2*x*=16) perennial legume forage, is a native grassland species widely distributed in hillsides, embankments, mixed grass steppes, and meadows of Siberia, Mongolia, and northern China [1]. It is also a close relative of alfalfa (*M*. *sativa*), which is the most important legume forage worldwide [2]. The distribution area of *M. ruthenica* is distinguished by dry, infertile soils, and long cold winter times [3]. Therefore, *M. ruthenica* must have evolved sophisticated mechanisms to survive the adverse environments, including drought, low temperatures, and infertile soil. Compared with other *Medicago* species, *M. ruthenica* is believed to be a relatively unique species among the *Medicago* species that are highly adapted to stressed environments, and whose potential application is positively evaluated in low-input systems, such as in the field without irrigation and in infertile soils [4].

Legume plants account for one third of primary crop yield as important sources for the consumption of human and animals in the world [5]. As the most important and popular legume forage, alfalfa has been cultivated in more than 80 countries with a total area of ~32 million hectares in the world [6]. Alfalfa is the top 10 crops in terms of cultivated area and is the third species only after soybeans and beans in legume crops (FAO, http://www.fao.org/faostat/en/#data/QC). In the major areas across the world, alfalfa is frequently exposed to unfavorable growth conditions, such as drought in Argentina and northern China, low temperature in Russian and Canada, saline/alkaline soils in California of America and Australia. Alfalfa yield and quality have been negatively impacted by environmental stress in these areas [7–10]. The great attention paid to the traits associated with high yield during long-term domestication of cultivated alfalfa may render the cultivated alfalfa less tolerant to harsh environments. Therefore, genetic resources rich in genes tolerant to harsh environments are eagerly required for breeding alfalfa varieties with high tolerance to environmental stresses. Despite assembly of the genomes for several legume crops, only four legume forages, *Lotus japonicas* [11], *M. truncatula* [12], red clover (*Trifolium pratense*) [13], and alfalfa [14, 15], have been genome-sequenced so far. Red clover is perennial legume forage with mild tolerance to low temperature and drought [13]. *L*. *japonicas* and *M*. *truncatula* are annual legume species with limited agronomic application [11, 12]. The *M*. *truncatula* genome can only provide limited information for alfalfa because it differs in lifecycle and pollination from alfalfa [16]. The whole-genome of alfalfa was recently sequenced using cultivated cultivars [14, 15]. However, the two alfalfa cultivars used for the whole-genome sequencing are of moderate tolerance to drought stress [17]. Numerous genes responsible for tolerance to environmental stress in the cultivated species may have been lost during domestication by selecting those traits associated with high yield and nutritional quality. Therefore, genomic information in the wild species can provide valuable clues for improving traits associated with adaptation to stressed environments in perennial legume forages.

As a close relative of alfalfa, *M. ruthenica* is also a perennial species with comparable genome size, life cycle, and pollination strategies to alfalfa. More importantly, as a wild species with many accessions occurring widely in the arid and/or semi-arid areas, it is highly tolerant to drought stress and has been used as parental materials to improve alfalfa tolerance to adverse environments by breeding alfalfa cultivars tolerant to environmental stress [2, 18]. Therefore, *M. ruthenica* can be a valuable model forge to study molecular mechanisms underlying tolerance to environmental stress in legume forages in general and alfalfa in particular. Moreover, comparative genomes of cultivated alfalfa with their wild relatives can identify the useful alleles in the wild species for trait improvement of cultivated alfalfa, thus contributing to our understanding of cultivated alfalfa’s domestication process. However, genomic information of *M. ruthenica* is scarce, which greatly hampers our molecular elucidation of stress physiology and its application to genomic improvements in tolerance of alfalfa to environmental stress. To facilitate genomic improvements of alfalfa in terms of enhanced tolerance to abiotic stress and understand the mechanisms underlying the great tolerance of *M*. *ruthenica* to environmental stress, we assembled and analyzed the genome of *M*. *ruthenica* that occurs naturally in arid and semi-arid areas of northern China. We further explored the mechanisms underlying the super tolerance of *M*. *ruthenica* to drought stress by an integrated approach. We firstly compared the transcription factor genes of *M. ruthenica* with *M*. *truncatula* and *M*. *sativa*. We further analyzed single-nucleotide polymorphism (SNP) variations and identified hundreds of candidate genes associated with drought tolerance using 20 accessions of *M. ruthenica* collected across different geographic sites in China. Numerous drought-responsive genes were identified by transcriptome, and their roles in the regulation of drought tolerance in the *M*. *ruthenica* were discussed.

## Results

### Genome sequencing and assembly

**Fig. 1:**
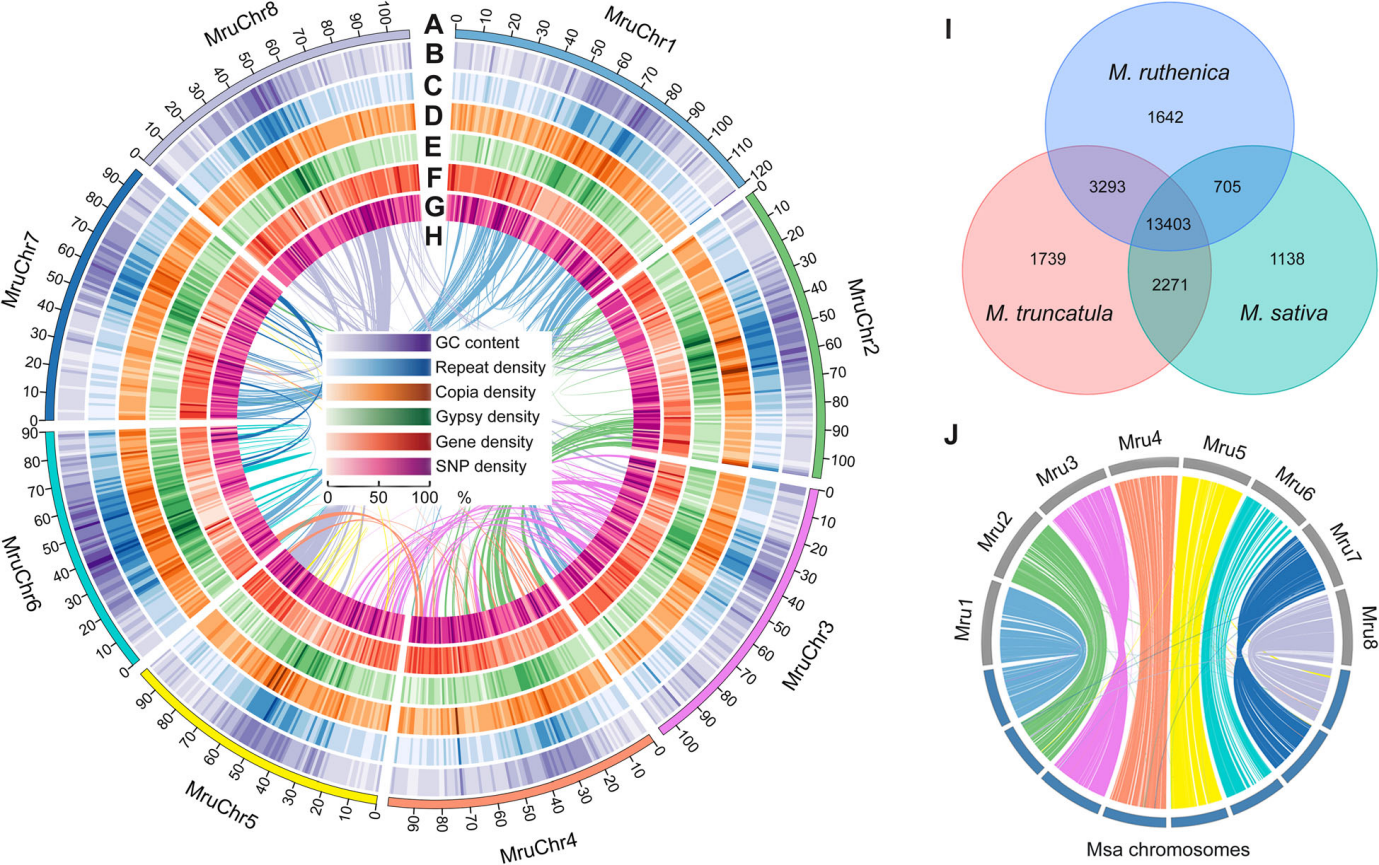
Distribution of genomic features within the *M*. *ruthenica* genome and genome comparison with *M*. *truncatula* and *M*. *sativa*. **a** Length bar of eight chromosomes (scale in 1 Mb). **b** GC content density. **c** Repeat density. **d** Copia density. **e** Gypsy density. **f** Gene density. **g** Single-nucleotide polymorphism density. **h** The synteny within *M*. *ruthenica* genome. **i** Shared and unique gene families in *M*. *ruthenica*, *M*. *truncatula*, and *M*. *sativa*. **j** Synteny between genomes of *M*. *ruthenica* and *M*. *sativa*. Mru *M. ruthenica*, Msa *M. sativa*

By K-mer analysis, the genome size and heterozygosity of *M. ruthenica* (Xinghe accession) were estimated to be about 914 Mb and 2.2%, respectively (Additional file 1: Figure S1, Additional file 2: Table S1). The genome was bigger than monoploid genome of cultivated alfalfa (~800 Mb) [14, 15] and much larger than that of *M*. *truncatula* (~500 Mb) [12]. We assembled 96.32 Gb PacBio single-molecule long reads (105×) and 301.44 Gb Illumina shotgun reads (330×) by sequencing paired-end and mate-pair libraries (250 bp-20 Kb). Moreover, 119.3 Gb data (131×) were obtained from the linked read-sequencing library by 10×Genomics platform to assist assembly (Additional file 2: Table S2). Using these data, we generated a Version 0.8 genome with the contig N50 size of 632.05 Kb and scaffold N50 size of 2.17 Mb mainly by the FALCON algorithm (Additional file 2: Table S3). To anchor the scaffolds to the chromosomes, 106.45 Gb high-throughput chromosome conformation capture (Hi-C) data (116×) were mapped to the Version 0.8 genome (Additional file 1: Figure S2). A total of 825.32 Mb representing 91.3% of the total assembled genome length was anchored to the eight chromosomes of *M. ruthenica* with the length of 90.05~120.35 Mb (Fig. 1a). The Version 1.0 genome was generated with the scaffold N50 size of 99.39 Mb, and GC proportion of 35.9% using LACHESIS (Table 1, Fig. 1b, Additional file 1: Figure S3, Additional file 2: Table S4). The completeness of genome assembly was evaluated by calculating the genome coverage rate (99.8%) to our assembled genome. The mapping rate of the Illumina paired-end reads and PacBio reads was 93.7% and 97.9%, respectively. Transcriptome data were also used to evaluate the completeness of genome assembly. The average 93.2% of transcriptomic reads was accurately mapped to the final genome assembly (Additional file 2: Table S5). The transcriptome assembly composing of 45,397 unigenes (>500 bp) was mapped to the genome assembly. More than 96.5% of these unigenes were identified in the *M. ruthenica* genome and 92.8% of them were covered more than 50% by one scaffold (Additional file 2: Table S6). Moreover, BUSCO analysis revealed that 91.3% of 1375 single-copy ortholog genes from the Embryophyta were complete in the genome assembly (83.2% as single-copy, 8.1% as duplicates). Conserved Core Eukaryotic Gene Mapping Approach (CEGMA) analysis revealed that 94.0% of the core protein-coding genes were identified in our assembled genome. These results suggest that the highly complete genome of *M. ruthenica* is assembled in the present study.

**Table 1:** The statistics of the *M*. *ruthenica* genome

Genome assembly		N50	N90	Total length
	Contigs	612.99 Kb	247.80 Kb	902.93 Mb
	Scaffolds	99.39 Mb	90.05 Mb	904.13 Mb
Genome annotation	Repetitive sequences	Categories	Length (bp)	Percentage (%)
		DNA transposon	24,361,896	2.6841
		LTR	470,694,297	51.859
		LINE	15,815,260	1.7425
		SINE	81,673	0.0090
		Other	6,173,813	0.6753
	Protein-coding genes	Number	Annotated number	Average CDS length
		50,162	49,176	1050 bp
	Non-coding RNAs	Categories	Number	Percentage (%)
		miRNA	3820	0.0538
		tRNA	788	0.0065
		rRNA	364	0.0050
		snRNA	2892	0.0355

*LTR* long terminal repeat, *LINE* long interspersed nuclear elements, *SINE* short interspersed nuclear elements, *CDS* coding sequence

### Genome annotation

Repetitive sequences comprised 57.0% in the genome of *M. ruthenica*, including the tandem repeat sequences and transposable elements (Fig. 1c, Additional file 2: Table S7). Long terminal repeat (LTR) retrotransposons were the most abundant transposable elements, comprising 51.9% of the whole genome, including Copia elements (21.0% of genome) and gypsy elements (27.1% of genome) (Fig. 1d, e, Additional file 1: Figure S4, Additional file 2: Table S8). In soybean, common bean, pigeonpea, and alfalfa genomes, LTR retrotransposons were also the most abundant transposable elements, representing 42.0%, 36.7%, 37.1%, and 27.4% of their genome size, respectively [19–21].

To predict protein-coding genes, we sequenced transcriptomes from roots, stems, leaves, flowers, and pods. In addition, we performed de novo and homolog-based predictions. The final reference gene set contained 50,162 protein-coding genes, with 3339 bp transcripts, 4.02 exons, and 1050 bp coding sequences on average (Fig. 1f, Additional file 1: Figure S5, Additional file 2: Table S9). The density of genes was correlated with that of single-nucleotide polymorphism (SNP) in some positions in chromosomes (Fig. 1g).

Of all the protein-coding genes in *M. ruthenica*, 49,176 genes (accounting for 98.0%) were annotated (Additional file 2: Table S10). The number of protein-coding genes in *M. ruthenica* genome was similar with that in *M*. *truncatula* and *M*. *sativa* [12, 15], but it was more than that in chickpea [22] and red clover [13] (Additional file 2: Table S11). After clustering, 27,764 gene families and 843 common single-copy orthologs were detected across *M. ruthenica* and six other species by OrthoMCL (Additional file 1: Figure S6). Of them, 13,403 gene families were identified as common among the three *Medicago* species (Fig. 1i). We further identified unique genes from the *M. ruthenica* genome, and their Gene Ontology (GO) terms were analyzed (Additional file 2: Table S12). Multiple enriched GO terms related to stress response were identified, including cellular response to stimulus (GO: 0051716), response to stress (GO: 0006950), response to stimulus (GO: 0050896), and response to wounding (GO: 0009611). In addition, two GO terms related to DNA replication (GO: 0006281, DNA repair; GO: 0006260, DNA replication) were enriched. These enriched GO terms suggest that there is a specific mechanism to resist stresses and repair DNA after damage, which may contribute to the tolerance of *M. ruthenica* to stresses. Moreover, we predicted 3820 microRNAs, 2892 snRNAs, 788 tRNAs, and 364 rRNAs in the *M. ruthenica* genome (Additional file 2: Table S13).

The genome size of *M*. *ruthenica* was about two times greater than that of *M*. *truncatula*. The repetitive sequence length in the genome of *M*. *ruthenica* was much longer than that of *M. truncatula* (517 Mb vs. 125 Mb) [12]. This may explain the greater genome size of *M. ruthenica* than that of *M. truncatula*. We further compared the density of transposable elements along the eight chromosomes between the two species and found that they were much more abundant in the genome of *M*. *ruthenica* (Additional file 1: Figure S7). Moreover, despite similar number of protein-coding genes in the two species (Table 1, Additional file 2: Table S11), the average length of transcripts in the *M*. *ruthenica* genome was longer than that in *M. truncatula* (3339 bp vs. 2332 bp).

### Genome evolution and synteny analysis

**Fig. 2:**
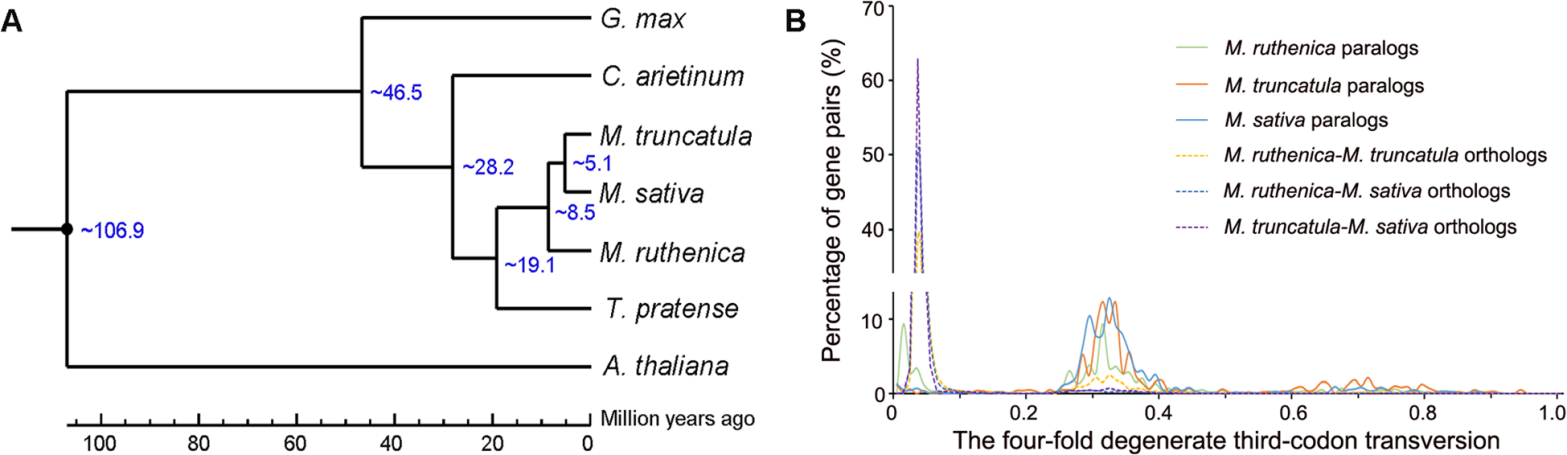
Phylogenetic tree, divergence time of seven species (**a**), and the four-fold degenerate third-codon transversion rate of gene pairs in three *Medicago* species (**b**). The phylogenetic tree was constructed based on 843 single-copy orthologous genes across *M. ruthenica* and the other six species by RAxML software, and the divergence times (Mya) are indicated by the blue numbers beside the branch nodes in panel **a**. The common whole-genome duplication events are indicated by the peaks (4DTv=0.32) in panel **b**

The abovementioned 843 common single-copy orthologs were used to construct the phylogenetic tree by RAxML software. In the Papilionoideae subfamily, the galegoid (*M*. *ruthenica*, *M*. *sativa*, *M*. *truncatula*, *C. arietinum*, and *L. japonicus*) and millettioid (*G. max*) clades separated ~46.5 millions of years ago (Mya). Within the galegoid clade, the split of *M. ruthenica* was estimated at ~8.5 Mya from *M. truncatula* and *M. sativa*, and the divergence from red clover and chickpea was estimated at ~19.1 Mya and at ~28.2 Mya, respectively (Fig. 2a).

We further constructed the genome synteny within and among *M*. *ruthenica*, *M*. *truncatula*, chickpea, and red clover by Mcscan software, respectively (Fig. 1h, Additional file 1: Figure S8, Additional file 1: Figure S9, Additional file 1: Figure S10, Additional file 2: Table S14). Most chromosomes in the *M*. *ruthenica* genome aligned mainly with their corresponding *M. truncatula* chromosomes. However, the chromosome 4 and 8 of *M*. *truncatula* exhibited translocation by analyzing its genome synteny with *M. ruthenica* and chickpea, suggesting that the chromosome rearrangements in the genome of *M*. *truncatula* have occurred after speciation (Additional file 2: Table S15). We also analyzed the genome synteny between *M*. *ruthenica* and *M*. *sativa* using the genome data of the most recent genome of alfalfa and cultivated alfalfa at the diploid level (Fig. 1j, Additional file 1: Figure S11). These results suggest that the two legume species are closely related in terms of genome sequence.

We extracted all the duplicated gene pairs from the syntenic blocks and calculated the four-fold degenerate third-codon transversion (4DTv) distance. *M*. *ruthenica*, *M*. *truncatula*, and *M*. *sativa* shared a whole genome duplication event (4DTv~0.32). The 4DTv distance of *M. ruthenica*-*M. truncatula* orthologs and *M. ruthenica*-*M. sativa* orthologs confirmed that the three species share a genome duplication event (Fig. 2b).

### Transcription factors and gene family expansion/contraction analysis

**Fig. 3:**
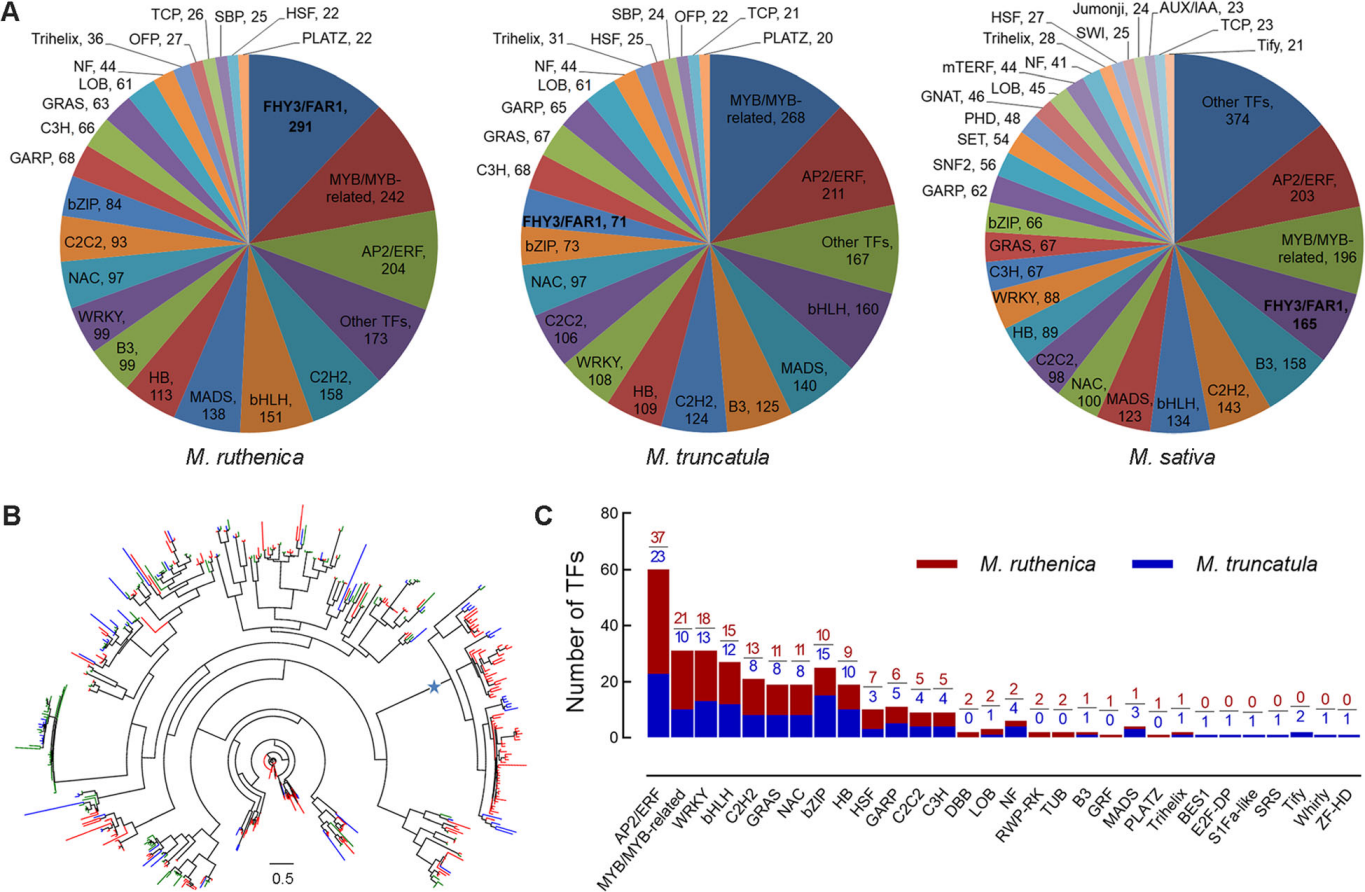
Transcription factors of *M*. *ruthenica*, *M*. *truncatula*, and *M*. *sativa*. **a** Transcription factor distribution of *M*. *ruthenica*, *M. truncatula*, and *M. sativa* in different families. Transcription factors of *M. ruthenica*, *M. truncatula*, and *M*. *sativa* were identified using iTAK software. All transcription factor families with less than 20 members were grouped into Other TFs. **b** Phylogenetic tree of FHY3/FAR1 family in *M*. *ruthenica* (red), *M*. *truncatula* (blue), and *M*. *sativa* (green). The scale bar in the tree shows the number of amino acid substitutions per site. The most significant cluster of *M*. *ruthenica* is marked by a star. Phylogenetic tree was drawn by FastTree software. **c** Comparison of drought-responsive transcription factor genes between *M. ruthenica* and *M. truncatula*

We identified 2402 TFs in *M*. *ruthenica* genome by comparing their sequences with the known domains of TFs using iTAK. The number of TFs accounted for 4.8% of the 50,162 protein-coding genes, and the TFs were distributed in 49 families. In addition, we identified 2207 TFs that belong to 49 families in the *M. truncatula* genome, and 2638 TFs that belong to 73 families in the *M. sativa* genome (Fig. 3a, Additional file 2: Table S16). The three species shared 47 common TF families. Far-red elongated hypocotyl 3/Far-red impaired response 1 family (FHY3/FAR1) is a positive transcription factor in the phytochrome A pathway. We found 291 *FHY3/FAR1* genes in the genome of *M. ruthenica*, which was the largest TF family in *M. ruthenica*. We checked the authenticity for all the 291 *FHY3/FAR1* genes one by one using Pacbio long reads by the IGV software and found all these genes were covered by Pacbio long reads. And the FPKM value (fragments per kilobase of exon model per million mapped fragments) of 175 *FHY3/FAR1* genes was more than 0.1 (Additional file 2: Table S17). The TF number in the most common families of the three species was comparable, and members of the *FHY3/FAR1* genes in *M. ruthenica* were greater than those in *M. truncatula* and *M. sativa* by 220 and 126, respectively. Phylogenetic tree of FHY3/FAR1 family in the three *Medicago* species showed expansion of this family in the *M. ruthenica* genome originated from several paralogs of *FHY3*/*FAR1* family (Fig. 3b). We found a significant cluster of *M. ruthenica* FHY3/FAR1 family in the phylogenetic tree, which consisted of 69 *M. ruthenica* branches, 13 *M. truncatula* branches, and 1 *M. sativa* branch. Moreover, 122 *FHY3*/*FAR1* genes occurred in the synteny blocks across the eight chromosomes in the *M*. *ruthenica* genome, and two genes (*FHY3/FAR1-200* and *FHY3/FAR1-201*) occurred in clusters, suggesting that *MruFHY3/FAR1* genes originate from both whole genome duplication and tandem duplication (Additional file 1: Figure S12; Additional file 2: Table S17). In contrast, no *FHY3*/*FAR1* genes were identified in clusters of the genome of *M. truncatula*, suggesting that *MtrFHY3/FAR1* genes only originate from whole genome duplication (Additional file 2: Table S18).

We also analyzed other expanded and contracted gene families (Additional file 2: Table S19, Additional file 2: Table S20). GO analysis of expanded families led to the enriched terms that were related to stress response (GO: 0006950, response to stress), signal transduction (GO: 0004965, G-protein-coupled GABA receptor activity), mineral element absorption (GO: 0008272, sulfate transport; GO: 0030955, potassium ion binding), and regulation of gene expression (GO: 0006306, DNA methylation; GO: 0003676, nucleic acid binding).

### Analysis of genome variation during domestication

The genome of *M. ruthenica* was used as a reference to analyze the genome variation of *M. truncatula* and *M*. *sativa* during domestication. A total of 1269 and 309 genes were generated in *M. truncatula* and *M*. *sativa* during domestication, respectively. Further, we found that 1954 and 579 genes were lost in *M. truncatula* and *M*. *sativa* during domestication, respectively. A total of 45 and 22 GO terms were enriched based on these lost genes in *M. truncatula* and *M*. *sativa*, respectively (Additional file 2: Table S21, Additional file 2: Table S22). Some GO terms related with stress tolerance were enriched for *M. truncatula* and *M*. *sativa*, such as cellular response to stimulus (GO: 0051716), nucleic acid binding (GO: 0003676), and DNA repair (GO: 0006281).

### Comparison of drought tolerance among legume forages

The wide occurrence of *M. ruthenica* in the arid and semi-arid areas prompts us to evaluate the molecular characteristics underlying its tolerance to drought. We compared the drought tolerance of *M. ruthenica* with that of *T. pretense*, *M. truncatula*, *M. varia*, *M. falcata*, and two alfalfa cultivars. Seedlings of *M. ruthenica* exhibited greatest tolerance to drought stress among the legume species examined (Additional file 1: Figure S13). For example, exposure of seedlings to drought led to marked reductions in survival rates for other legume forages, while the same treatment had little effect on survival rate of *M. ruthenica* seedlings, highlighting the strongest tolerance of *M. ruthenica* to drought stress among the legume forages examined. Previous studies have shown that the drought tolerance of *M*. *varia* and *M. falcata* was greater than the two alfalfa cultivars used for genome-sequencing [17]. Therefore, the drought tolerance of *M. ruthenica* is much stronger than that of the two alfalfa cultivars.

To identify the potentially key genes underlying the drought tolerance, root samples of genome-sequenced *M. ruthenica* (Xinghe accession) exposed to drought stress for varying times were used to construct cDNA libraries. High-throughput sequencing (RNA-seq) of 12 libraries led to generation of 84.90 G clean data (Additional file 2: Table S23). Drought-responsive TF genes were identified by comparing the normalized expression levels of genes between libraries of drought stress and control using DESeq. We identified 183 TF genes that were responsive to drought at the 7th day of drought treatment (Additional file 2: Table S24), while 144 drought-responsive TF genes were detected in *M. truncatula* under the identical drought regime (Fig. 3c, Additional file 2: Table S25). Furthermore, we found that drought-responsive members of AP2/ERF family were the most abundant in both species. For example, we detected 37 and 23 drought-responsive TF genes of AP2/ERF family in *M. ruthenica* and *M. truncatula*, respectively. Of MYB/MYB-related family, we identified 21 and 10 drought-responsive TFs in *M. ruthenica* and *M. truncatula*, respectively. In addition, several drought-responsive TF families, including WRKY, bHLH, C2H2, GRAS, NAC, bZIP, and HB, were markedly regulated in the two species under drought stress (Fig. 3c).

### Selective sweep analysis among different accessions of *M. ruthenica*

**Fig. 4:**
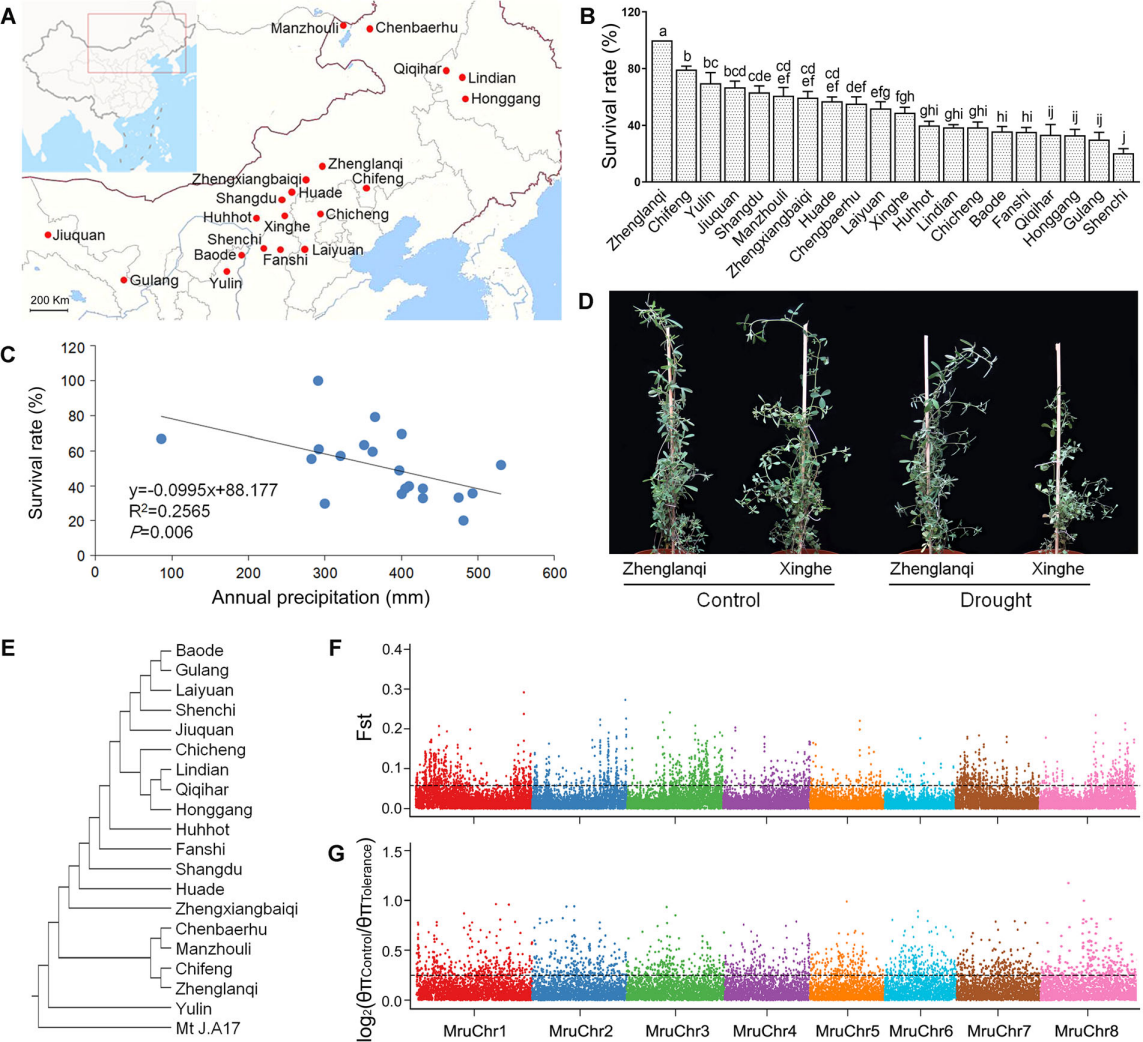
Comparison of tolerance to drought stress, population differentiation, and nucleotide diversity ratio among different *M. ruthenica* accessions. **a** The geographic sites for collection of 20 *M. ruthenica* accessions. **b** Survival rates of 20 *M. ruthenica* accessions at the twelfth day exposed to drought stress. Four biological replications and 15 seedlings in each biological replication were used to determine survival rate. All the survival rates under control are 100%. Different letters mean significant differences among treatments at *P*<0.05. Data are means±SE (*n*=4). **c** Correlations between survival rates under drought stress and annual precipitation of sites at which the *M. ruthenica* seeds were collected. **d** Phenotypes of two accessions collected from sites of Zhenglanqi and Xinghe. The phenotypes were photographed at the twelfth day of drought. **e** Neighbor-joining tree of *M. ruthenica* accessions. Mt J.A17: *M. truncatula* Jemolong A17. **f** Population differentiation (Fst). **g** Nucleotide diversity ratio (log_2_(θπ_Control_/θπ_Tolerance_)) between tolerance and control population. Points above lines are 5% of the biggest Fst and log_2_(θπ_Control_/θπ_Tolerance_), respectively. Points of log_2_(θπ_Control_/θπ_Tolerance_) less than zero are not shown

Given the wide distribution of *M. ruthenica* in China [23], we compared tolerance of different accessions to drought stress by collecting seeds of *M. ruthenica* 20 accessions from different locations across northern China (Fig. 4a, b). We found significant correlations between the survival rates of the 20 *M. ruthenica* accessions under the drought stress and annual precipitation at sites where their seeds originally collected (Fig. 4c). This result suggests that tolerance to drought may result from evolutionary adaptation of *M. ruthenica* to the arid environments. Of the 20 *M. ruthenica* accessions, the Zhenglanqi accession exhibited the highest survival rate under drought stress (Fig. 4b, d).

To identify single-nucleotide polymorphism (SNP) variation associated with drought tolerance among different *M. ruthenica* accessions, we performed whole genome resequencing of 19 accessions in addition to Xinghe accession that was used for the whole-genome sequencing. We obtained a total of 222.84 G clean data, and their 13× average depth for the 19 accessions (Additional file 2: Table S26). We obtained a total of 14,212,747 SNPs with high quality (Additional file 2: Table S27). A rooted phylogenetic tree was constructed using the SNP information by neighbor-joining method (Fig. 4e), and principal component and population structure were also analyzed (Additional file 1: Figure S14; Additional file 1: Figure S15). In addition, according to the survival rates of the 20 accessions under drought stress (Fig. 4b), we grouped the top 7 tolerant accessions as the tolerant population, while the least 7 tolerant accessions were set as the control population. The population differentiation (Fst) and nucleotide diversity ratio log_2_(θπ_Control_/θπ_Tolerance_) were calculated using SNP data across the 40 kb windows with a 20 kb slide (Fig. 4f, g, Additional file 1: Figure S16). These allowed us to identify 367 candidate genes associated with drought tolerance (Additional file 2: Table S28). GO analysis led to enriched terms related with photosynthesis (GO: 0019684, photosynthesis, light reaction; GO: 0022900, electron transport chain; GO: 0003843, 1,3-β-d-glucan synthase activity) and stress response (GO:0006979, response to oxidative stress; GO: 0004601, peroxidase activity) (Additional file 2: Table S29).

### Transcriptome analysis

**Fig. 5:**
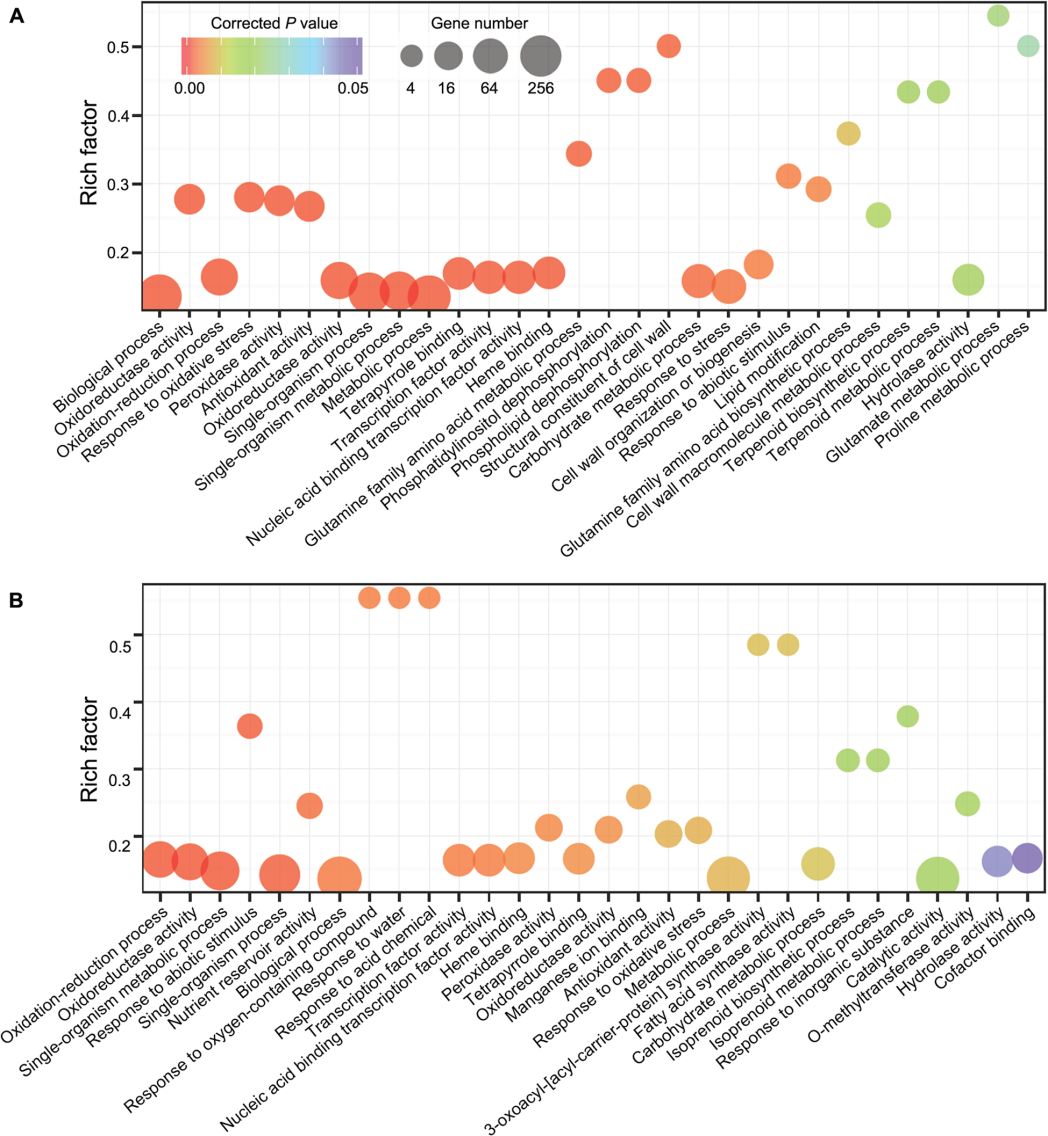
The GO enrichments of drought-responsive genes at the 7th day of drought treatment from the two *M. ruthenica* accessions. **a** Zhenglanqi accession. **b** Xinghe accession. The significant drought-responsive genes and enriched GO terms were identified using a corrected *P*<0.05. Rich factor is the proportion of the differentially expressed gene number to the total gene number in a given GO term. The top 30 GO terms were shown in the figure

To explore the regulatory mechanisms of gene expression under drought stress, the most drought tolerant Zhenglanqi accession was selected to perform transcriptome analysis, with the Xinghe accession whose genome had been assembled in this study as control. Root samples treated with drought stress of varying times were used to construct cDNA libraries. High-throughput sequencing (RNA-seq) of 24 libraries led to generation of 168.29 G clean data (Additional file 2: Table S23). Drought-responsive genes were identified by comparing the normalized expression levels of genes from drought stress and control libraries using DESeq. Exposure to drought stress led to alterations in expression patterns of 1383 and 1693 genes in roots of the accessions collected from sites of Zhenglanqi and Xinghe, respectively. The number of drought-responsive genes peaked at the 7th day among different treatments of stringency (Additional file 1: Figure S17). A network of drought-responsive genes was constructed using STRING (Additional file 1: Figure S18), and the GO terms of drought-responsive genes were enriched (Additional file 2: Table S30, Additional file 2: Table S31). Taken the 7th day of drought stress as an example, we found that the Zhenglanqi accession exhibited greater enrichments of GO terms compared to Xinghe accession (79 vs. 30 GO terms) and that the GO terms in molecular functions (GO:0016209, antioxidant activity; GO:0001071, nucleic acid binding transcription factor activity) and biological processes (GO:0006979, response to oxidative stress) were enriched in the two accessions (Fig. 5).

## Discussion

*Medicago ruthenica* is a wild legume forage with super tolerant to varying abiotic stresses. Therefore, it provides a valuable genetic resource for improving traits associated with tolerance to environmental stress in legume forage in general and in alfalfa in particular [3, 24]. However, the lack of reference genome for the perennial legumes with super tolerance greatly hampers our molecular understanding how perennial legume forages respond and adapt to the harsh environments. To fill this gap, we assembled a chromosome-scale genome of *M*. *ruthenica* in high quality using 623.51 Gb data (682× of genome size). To the best of our knowledge, our genome sequence is the first one for a wild legume forage species with great tolerance to environment stress.

The ancestors of all flowering plants have experienced the whole genome duplication (WGD), which is an important evolutionary force for speciation, adaptation, and diversification [25, 26]. Moreover, the WGD event often occurs in the ancestors of the species-rich groups, including legumes [27]. The analysis of *M. truncatula*, *G. max*, and other Papilionoid genomes of legumes characterized a common WGD event in the Papilionoideae subfamily [12, 20]. To unravel the genome evolution, we constructed the genome synteny among *M. ruthenica*, *M. truncatula*, and alfalfa (Fig. 1i, Additional file 1: Figure S8). The analysis of 4DTv distance revealed that the three species shared the ancestral Papilionoideae WGD (Fig. 2b). We identified 2977 orthologous genes between *M. ruthenica* and *M. sativa* from their own paralogous blocks (Additional file 1: Figure S19). This result illustrates that *M. ruthenica* and *M. sativa* shares the ancestral Papilionoideae WGD and have undergone a similar evolutionary course. The left genes present in the paralogous blocks were retained in the different species during genome recombination after the WGD or generated after speciation. These genes may contribute to the origin of species specificity.

**Fig. 6:**
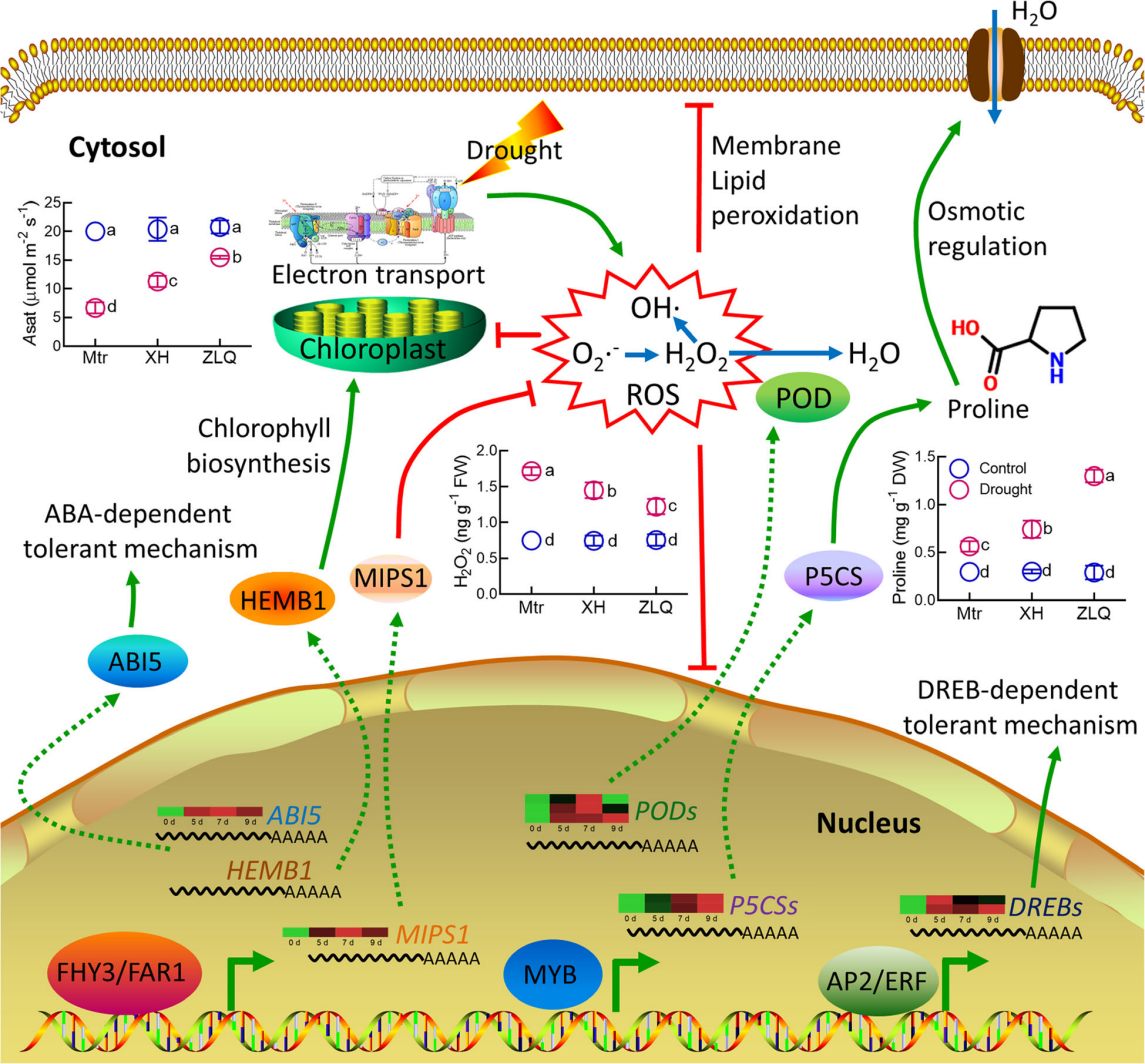
Proposed mechanisms behind tolerance of *M. ruthenica* to drought stress. The dotted lines represent the transfer and translation. Arrows denote positive effects, whereas lines ending with a short bar indicate negative effects. Photosynthetic rates were measured using the newly expanded leaves treated for 7 days by a gas exchange system (LI-COR 6800). Root samples at the seventh day of drought were used to determine H_2_O_2_ and proline. *A*sat photosynthetic rate, ROS reactive oxygen species, Mtr *M. truncatula*, XH Xinghe accession, and ZLQ Zhenglanqi accession. Data are means±SE (*n*=4). Different letters mean significant differences among treatments at *P*<0.05

The gene family expansion can enhance adaptations to changing environments [28]. FHY3/FAR1 family in *M. ruthenica* was the largest among those in the galegoid clades of Papilionoideae subfamily according to the known genome information. FHY3/FAR1 family has been suggested to directly activate the expression of *HEMB1* and *ABI5*, which regulates chlorophyll biosynthesis [29], and ABA-dependent tolerance to abiotic stress [30], respectively (Fig. 6, Additional file 1: Figure S20A). Furthermore, FHY3/FAR1 can detoxify ROS by upregulating expression of *MIPS1* to enhance the biosynthesis of inositol [31]. This may explain the higher expression of *MrMIPS1* and less accumulation of in H_2_O_2_ in *M. ruthenica* than in *M. truncatula* under drought stress (Fig. 6, Additional file 1: Figure S20B). The large-scale expansion of FHY3/FAR1 family may confer *M. ruthenica* great tolerant to abiotic stress among the legume forages examined in our study. In addition, our results also reveal that AP2/ERF family, including DREB TFs, may contribute to the most drought-responsive members in *M. ruthenica* and *M*. *truncatula* (Fig. 3c, Additional file 1: Figure S20C).

Traits associated with high yield have been paid great attention during the process of domestication, thus rendering the cultivated alfalfa less tolerant to environmental stress due to the loss of traits involved in adaptation to harsh environments [32]. We identified the lost genes in cultivated *M. truncatula* and *M*. *sativa* during domestication (Additional file 2: Table S22, Additional file 2: Table S23). More genes were lost in *M. truncatula* compared with *M*. *sativa*. Most enriched GO terms based on lost genes in *M*. *sativa* during domestication were also enriched in *M*. *sativa*. In the GO term of “nucleic acid binding,” *FHY3/FAR1* genes were identified, which is consistent with the transcription factor analysis among the three species (Fig. 3a). In addition, we found that *DNA helicase* genes represented high proportion in the GO term “DNA repair.” This gene family codes for molecular motor proteins in various cellular mechanisms and regulators in the pre-mRNA splicing and plays important roles in alleviating multiple abiotic stresses [33]. The *Helicase 45* of pea is responsive to salinity, dehydration, and low temperature. Expression of this gene confers transgenic plants more tolerance to abiotic stress [34–36]. It is expected that the lost genes associated with stress response, transcriptional regulation, and DNA repair may explain the less tolerance of two cultivated species than wild *M. ruthenica*.

The great variations in drought tolerance among the *M. ruthenica* accessions allowed us to explore mechanisms underlying the tolerance of *M. ruthenica* to drought. The identified candidate genes associated with drought tolerance were involved in photosynthesis and stress response (Additional file 2: Table S29). The photosynthetic electron transport chain is suppressed under drought stress, thus leading to massive accumulation of ROS. Accessions of *M. ruthenica* with a greater drought tolerance may be equipped with more efficient photosynthesis systems, thus allowing *M. ruthenica* to supply energy by maintaining relatively high photosynthetic rates under drought stress (Fig. 6).

We further explored the mechanisms underlying the greater tolerance of Zhenglanqi accession than that of Xinghe accession by a transcriptomic approach. The two closely correlative GO terms with drought tolerance (GO: 0006950, response to stress; GO: 0006560, proline metabolic process) were enriched in the Zhenglanqi accession, but not in the Xinghe accession (Fig. 5, Additional file 2: Table S30, Additional file 2: S31). Moreover, the common GO terms of nucleic acid-binding transcription factor activity in Zhenglanqi accession showed higher degree of enrichment. To identify the potential target genes of TFs in the two accessions, we analyzed the promoter elements of all the responsive-genes. We identified the involvements of the target genes of MYB TFs in detoxification (e.g., *POD*) and proline synthesis (e.g., *P5CS*). The less accumulation of H_2_O_2_ and greater accumulation of proline in the Zhenglanqi accession may be accounted for by the GO enrichment of peroxidase activity and proline biosynthetic process (Fig. 6, Additional file 1: Figure S20D, Additional file 1: Figure S20E, Additional file 2: Table S32). Moreover, the observation that more target genes of MYB in the GO terms of the Zhenglanqi accession may suggest important roles of MYB transcription factors in drought tolerance of *M. ruthenica* (Additional file 2: Table S32).

*Medicago ruthenica* has capacious prospects to provide resource for improving traits associated with tolerance to environmental stress in alfalfa. The alfalfa genome will greatly push this development [14, 15]. The assembly of *M*. *ruthenica* genome opens a new avenue for genomic- and genetic-based improvement of legume forage, particularly for elucidation of molecular mechanisms associated with response and adaptation to environmental stress for alfalfa. In the present study, we also identified lost genes in cultivated alfalfa and *M. truncatula* during domestication, and stress-selective/responsive genes in *M. ruthenica*, which can be used in the tolerant trait improvement of cultivated alfalfa. Induced distant hybridization of alfalfa and *M. ruthenica* may be an effective way to breed new cultivated species. Innovative breeding technology can be used in breeding alfalfa with great tolerance to harsh environments. Recent advances in CRISPR/Cas genome editing enable effectively targeted modification in most crops, thus accelerating crop improvement [37]. The specifically expressional genes in *M. ruthenica* under stress and the lost genes of alfalfa during domestication can be candidate genes for transforming alfalfa using CRISPR/Cas9 system for breeding tolerant varieties. The genome information and identification of resistance genes of *M. ruthenica* can shed important light on the mechanisms behind the tolerance of *M. ruthenica* to environmental stress and provide valuable resource for improvements of agronomic traits associated with high yield and great tolerance to environmental stress through a molecular breeding approach.

## Conclusions

In this study, we assembled a high-quality reference genome for a perennial legume forage of *M. ruthenica* and explored the molecular mechanisms by which *M. ruthenica* adapts to the drought stress using genomic and genetic approaches. Specifically, we analyzed transcription factors, expanded/contracted gene families, and retained genes compared with cultivated Medicago species, SNP variations, and drought-responsive genes by comparative genomics, resequencing, and transcriptomics. Therefore, the genome of *M. ruthenica* provides new insights into the tolerant mechanisms of legume forage to environmental stress and valuable information for genetic-based improvements of agronomic traits in perennial legume crops.

## Methods

### Sequencing and assembly

Leaf samples from a single plant of *M. ruthenica* collected from Xinghe County (40° 56′ 2′′ N, 113° 31′ 4′′ E) in northern China were used for the genome sequencing. This accession of *M. ruthenica* is of a typical phenotype and distributed widely in China. A total of 76.54 Gb of reads from 270 bp and 500 bp insert size libraries were used to calculate the k-mer frequency distribution. The 17-mer sequences of sliding windows were used to estimate *M. ruthenica* genome size (the total number of k-mer/the depth of the major peak). The heterozygosity ratio of the *M. ruthenica* genome was estimated using GenomeScope based on the 17-mer data (http://qb.cshl.edu/genomescope/). De novo assembly of PacBio SMRT reads was performed using FALCON [38]. Thereafter, Pilon was used to perform the second round of error correction with the Illumina paired-end reads [39]. The Purge Haplotigs (https://bitbucket.org/mroachawri/purge_haplotigs) were used to remove the redundant sequences caused by heterozygosity. The scaffolding was performed by 10×Gscaff v2.1 using 10×Genomics data [40]. SSPACE v3.0 was used to build scaffolds using Illumina data from all the mate-pair libraries [41]. The assembly was used as input for scaffolding by fragScaff along with an N-base bed file and a repeat bed file produced by self-against-self BlastN [42]. The Hi-C data were mapped to the original scaffold genome using BWA v0.7.7 [43], and only reads with unique alignment positions were extracted to construct a chromosome scale assembly using LACHESIS [44]. The completeness of the assembly was assessed using BUSCO and CEGMA [45, 46].

### Annotation

Repetitive sequences include transposable elements (TEs) and tandem repeats. RepeatMasker v3.3.0 [47] was performed to detect TEs by comparing sequences with integrate repeat libraries including known repeat library (Repbase 15.02) and the de novo repeat library built by RepeatModeler v1.0.5, RepeatScout3, and LTR-Finder4. Tandem repeats were ascertained in the genome using Tandem Repeats Finder [48]. De novo predictions, homolog-based, and RNA-seq-based predictions were employed to annotate the protein-coding genes. Five ab initio gene prediction programs were used to predict genes, including Augustus v3.0.2, Genescan v1.0, Geneid, GlimmerHMM v3.0.2, and SNAP. Protein sequences of ten homologous species *A. thaliana*, *M. truncatula*, *G. max*, *T. pratense*, *C*. *arietinum*, *Vigna angularis*, *V. radiate*, *C. cajan*, *P. vulgaris*, and *Arachis ipaensis* were downloaded from the Ensembl or NCBI. Homologous sequences were aligned against to the repeat-masked *M. ruthenica* genome using TblastN (*e* value ≤1e^−5^). Genewise v2.2.0 was employed to predict gene models based on the alignment sequences [49]. There were two ways to assemble the RNA-seq data into the unique sequences of transcripts. One was mapping the RNA-seq data to the *M. ruthenica* genome using Tophat v2.0.8 [50] and Cufflinks v2.1.1 [51] for transcript assembly. The other was applying Trinity [52] to assemble the RNA-seq data, and then PASA software [53] improved the gene structures. A weighted and non-redundant gene set was generated by EVidenceModeler [54] which merged all genes models predicted by the above three approaches. PASA adjusted the gene models generated by EVM. Lastly, the gene sets were filtered according to the following standards: coding region lengths of amino acids ≤50, supported only by de novo methods and with FPKM<5. Functional annotation of protein-coding genes was obtained according to best Blast hit by BlastP (*e* value ≤1e^−5^) against SwissProt [55] and NCBI non-redundant (NR) protein databases. Motifs and domains were annotated by using InterProScan v4.7 [56] to search against InterPro v29.0 databases [56], including Pfam, Prints, Prosite, ProDom, and Smart. The tRNA genes were predicted by tRNAscan-SE software [57]. The miRNA and snRNA fragments were identified by INFERNAL software [58] against the Rfam database [59]. The rRNAs were found by using BlastN (*e* value ≤1e^−10^) against invertebrate rRNA database. The structure figure was drawn along eight chromosomes of *M. ruthenica* genome using Circos program [60]. To estimate the assembly of genome, transcriptome data from roots, stems, leaves, flowers, and pods were mapped to the genome assembly using Hisat2 [61], and transcripts were assembled using Trinity [52]. The unigene, which was the longest transcript selected from Trinity, was aligned to the genome assembly by Blat [62].

### Genome comparison and evolution

Gene families were generated by OrthoMCL [63]. Nucleotide and protein data of the other six species were downloaded from the Ensembl or NCBI. We selected the first group of allelic chromosomes (chr1.1–chr1.8) of *M. sativa* for analysis [14]. Before an “all against all” BlastP (*e* value ≤1e^−7^) program, the longest transcript was selected from alternative splicing transcripts belonging to one gene. The alignments with high-scoring segment pairs were conjoined for each gene pair by Solar [64]. To identify homologous gene-pairs, more than 30% coverage of the aligned regions was required. Finally, the alignments were clustered into gene families using OrthoMCL with 1.5 inflation index. GO enrichment of unique gene families in *M. ruthenica* were analysed. The shared single-copy orthologs were utilized to construct the phylogenetic tree. Protein sequences of these orthologs were aligned by muscle [65], then protein alignments were transformed to coding sequence (CDS) alignments. The phylogenetic tree was constructed by the ML (maximum likelihood) TREE algorithm in RAxML v7.2.3 [66]. The mcmctree program of Paml [67] was applied to estimate divergence time among 7 species. One calibration point was selected as normal priors to restrain the age of the nodes (89.8–125 Mya for *A. thaliana-G. max*). Mcscan [68] was used to construct the genome synteny within *M. ruthenica*, and among species of *M. truncatula*, *C. arietinum*, *T. pretense*, and *M. sativa*, respectively. We selected the first group of allelic chromosomes (chr1.1–chr1.8) of *M. sativa* for this analysis [14]. Syntenic blocks containing at least 5 genes were obtained based on the similarity gene pairs (Blastp: *e* <1e^−5^). We extracted all the duplicated gene pairs from syntenic blocks and calculated the 4DTv (transversion substitutions at fourfold degenerate sites) distance. The genome synteny between *M. ruthenica* and cultivated alfalfa at the diploid level (https://legumeinfo.org/data/public/Medicago_sativa/CADL_HM342.gnm1.rVNY/) was constructed using Nucmer (http://mummer.sourceforge.net/). CAFE was used to analyze gene family expansion [69]. Transcription factors of *M. ruthenica*, *M. truncatula*, and *M*. *sativa* were identified using iTAK software [70]. Phylogenetic tree of FHY3/FAR1 family was drawn by FastTree software.

The genome of *M. ruthenica* was used to analyze the genome variation of *M. truncatula* and *M*. *sativa* during domestication. The gene pairs between reference and target species were obtained according to best hit by BlastP (*e* value ≤1e^−5^). These gene pairs were sorted by linear order along chromosomes in the genome of target species. If there was one gene in the genome of target species, but no corresponding gene in the reference genome, the absent gene in the reference genome was marked “NA”. The absent region was defined as the upstream and downstream of “NA”. We used Exonerate software [71] to predict homolog-based gene on this region. If the genes of the target species were not predicted in the genome of reference species, it indicated that the genes of target species were specifically retained genes, and these genes were lost in the reference species.

### Survival rate and experiments of physiology

Seedlings of *T. pretense*, *M. ruthenica*, *M. truncatula*, *M. sativa*, *M. varia*, and *M. falcata* were grown in pots (7×7×12 cm) filled with vermiculite and peat soil (2:1) under controlled conditions (26°C day/20°C night, 16 h photoperiod). Drought stress was initiated by withholding water supply to 6-week-old seedlings for varying periods after seedlings were fully watered. In the comparison of *M. ruthenica* with other legume species, the phenotypes were photographed at the tenth day of drought. The survival rate at the tenth day of drought treatment was determined after re-watering for 7 days. In the comparison of 20 *M. ruthenica* accessions from different locations, the phenotypes were photographed at the twelfth day of drought, and survival rate at the twelfth day of drought was counted using the identical protocols. Four biological replications and 15 seedlings in each biological replication were used to determine survival rate. Photosynthetic rates were measured using the newly expanded leaves treated for 7 days by a gas exchange system (LI-COR 6800). Air flow rate and CO_2_ concentration were maintained at 750 μmol s^-1^ and 400 μmol mol^-1^, respectively. Root samples at the seventh day of drought were used to determine H_2_O_2_ and proline [72, 73].

### Analysis of SNPs

Leaf samples of 19 accession of *M. ruthenica* were used for resequencing. Genomic DNA was isolated from these materials. Randomly fractured DNA was used to construct libraries using TruSeq Library Construction Kit (Illumina), and raw data were obtained using Illumina Hiseq PE150. The adapter sequences of the raw reads were trimmed, and low-quality reads were filtered to obtain clean data. Clean data were mapped to the genome of *M. ruthenica* using BWA [43]. The high-quality SNPs were screened using SAMTools [74]. SNPs were annotated using ANNOVAR [75]. Phylogenetic tree was constructed by neighbor-joining method with bootstrap values from a minimum of 1000 trials using TreeBeST software. According to the survival rate under drought stress, the top 7 tolerant accessions (Zhenglanqi, Chifeng, Yulin, Jiuquan, Shangdu, Manzhouli, and Zhengxiangbaiqi) were grouped to tolerance population, and the least 7 tolerant accessions were (Chicheng, Baode, Fanshi, Qiqihar, Honggang, Gulang, and Shenchi) grouped to control population. The population differentiation (Fst) [76] and nucleotide diversity (θπ) [77] were calculated using SNP data across 40 kb windows with a 20-kb slide. The SNP windows were screened using top 5% Fst and log_2_(θπ_Control_/θπ_Tolerance_). The genes in these windows were identified to perform GO enrichment [78].

### Identification and analysis of drought-responsive genes

Root samples of *M. ruthenica* accessions from the sites of Zhenglanqi and Xinghe at the 5th, 7th, and 9th day under drought were harvested to construct transcriptome libraries. Three biological replicates were used for each treatment. The mRNA enriched from total RNA was used to construct RNA-seq libraries using TruSeq RNA Sample Preparation Kits. The libraries were sequenced on an Illumina Hiseq PE150 platform and paired-end reads (2×150 bp) were generated. The adapter sequences of the raw reads were trimmed, and low-quality reads were filtered to obtain clean data. Differential expression analysis was performed using the DESeq R package [79]. The resulting *P* values were adjusted using the Benjamini and Hochberg’s approach for controlling the false discovery rate. Genes with an adjusted *P* value <0.05 by DESeq were assigned as differentially expressed. From the data of Xinghe accessions at the 7th day under drought stress, drought-responsive TF genes were identified and drought-responsive TF genes of *M. truncatula* under the same treatment were identified using the published data [80]. STRING v11 [81] were used to build protein association networks, and figures were drawn by Cytoscape [82]. The GO terms of drought-responsive genes were analyzed in two accessions. Rich factor is the proportion of the differentially expressed gene number to the total gene number in a certain GO term. The protein sequence alignment was performed by RAxML with homologous proteins of other species [66]. The *cis*-acting regulatory elements were searched by PLACE [83]. The drought-responsive potential MYB-target-genes with more than two MYB-core elements were analyzed for both accessions using GO.

## Supplementary Information

**Additional file 1: Figure S1.** Distribution of 17-mer frequency and heterozygosity estimate of *M. ruthenica* genome. **Figure S2.** Hi-C interactome within and among chromosomes (Mru1-Mru8). **Figure S3.** Distribution of GC content and depth. **Figure S4.** Divergence distribution of classified families of transposable elements. **Figure S5.** Comparison of gene parameters among *Medicago ruthenica* genome and other genomes. **Figure S6.** The distribution of genes in different species. **Figure S7.** The density comparison of transposable elements between *M. ruthenica* and *M. truncatula*. **Figure S8.** Genome synteny between *M. ruthenica* and *M. truncatula*. **Figure S9.** Synteny analysis between genomes of *M. ruthenica* and *C. arietinum.* **Figure S10.** Synteny analysis between genomes of *M. ruthenica* and *T. pretense*. **Figure S11.** Synteny between *M. ruthenica* and *M. sativa* in the level of scaffolds. **Figure S12**. Distribution of *FHY3/FAR1* genes and synteny analysis within *M. ruthenica* genome. **Figure S13.** The survival rate comparison of other legume forages and *M. ruthenica* under drought stress. **Figure S14.** Principal component analysis plot of genetic variation. **Figure S15.** The genetic structure of populations. **Figure S16.** Selective sweep analysis using Fst & θπ. **Figure S17.** Venn diagram of drought-responsive genes from two *M. ruthenica* accessions collected from sites of Zhenglanqi and Xinghe. **Figure S18.** The predicted interaction of proteins encoded by drought-responsive genes in *M. ruthenica*. **Figure S19.** The number of genes present in the duplicated paralogous blocks of *M. ruthenica* and *M. sativa*. **Figure S20.** The expression of *MrABI5*, *MrMIPS1*, *MrDREBs*, *MrPODs* and *MrP5CSs* in two accessions under drought stress.

**Additional file 2: Table S1.** Estimation of genome size of *M. ruthenica* using 17-mer analysis. **Table S2.** Sequencing data generated from different platforms/strategies. **Table S3** The assemblage summary of the *M. ruthenica* genome Version 0.8 before chromosome anchoring by Hi-C. **Table S4.** The assemblage summary of the *M. ruthenica* genome Version 1.0. **Table S5.** Genome estimation using RNA sequencing data. **Table S6.** Assessment of the transcript coverage with the transcritome assembly. **Table S7.** The prediction of repeat elements in the *M. ruthenica* genome. **Table S8.** Categories of transposable elements predicted in the *M. ruthenica* genome. **Table S9.** General statistics of predicted protein-coding genes in *M. ruthenica.* **Table S10.** General statistics of mapping rate to functional database of protein-coding genes. **Table S11.** General statistics of protein-coding genes of homolog species. **Table S12.** GO enrichment of unique genes in *M. ruthenica*. **Table S13.** General statistics of non-coding RNA of the genome. **Table S14.** Statistics of synteny blocks within *M. ruthenica* and *M. truncatula*, *M. ruthenica* and *T. pratense*, *M. ruthenica* and *C. arietinum*, and *M. ruthenica* itself. **Table S15.** The corresponding relationship of synteny blocks in *M. ruthenica* with *M. truncatula.* ***T*able S16.** Statistics of TFs in *M. ruthenica*, *M. truncatula* and *M. sativa.* ***T*able S17.** Information of *FHY3/FAR1* genes in *M. ruthenica.* **Table S18.** Information of *FHY3/FAR1* genes in *M. truncatula*. **Table S19.** The GO enrichments of expanded families. **Table S20.** The GO enrichments of contracted families. **Table S21.** The GO enrichments of lost genes in the genome of *M. truncatula* during domestication. **Table S22.** The GO enrichments of lost genes in the genome of *M. sativa* during domestication. **Table S23.** Statistics of transcriptome libraries. **Table S24.** TFs responsive to drought in *M. ruthenica.* **Table S25.** TFs responsive to drought in *M. truncatula.* **Table S26.** Resequencing data of 19 accessions. **Table S27.** Statistics and annotation of SNPs. **Table S28.** Candidate genes associated with drought tolerance analyzed by selective sweep. **Table S29.** The GO enrichments of candidate genes associated with drought tolerance analyzed by selective sweep. **Table S30.** The GO enrichments of drought-responsive genes from Zhenglanqi accession. **Table S31.** The GO enrichments of drought-responsive genes from Xinghe accession. **Table S32.** Classification of potential MYB target genes which were responsive to drought stress.

## Data Availability

The assembled genome, transcriptome, and resequencing data have been deposited in GenBank under accession WNNG00000000 [84], PRJNA589938 [85] and PRJNA598783 [86], respectively. The files of genome assembly and annotation have also been deposited in figshare (10.6084/m9.figshare.12726932) [87].
