# Supplementary material for: The genome of a wild Medicago species provides insights into the tolerant mechanisms of legume forage to environmental stress
Source: BMC Biol. 2021 May 6;19:96. doi: 10.1186/s12915-021-01033-0 (PMC8103640; doi:10.1186/s12915-021-01033-0)
Supplement: Supplementary file 1 — Additional file 1: Figure S1. Distribution of 17-mer frequency and heterozygosity estimate of M. ruthenica genome. Figure S2. Hi-C interactome within and among chromosomes (Mru1-Mru8). Figure S3. Distribution of GC content and depth. Figure S4. Divergence distribution of classified families of transposable elements. Figure S5. Comparison of gene parameters among Medicago ruthenica genome and other genomes. Figure S6. The distribution of genes in different species. Figure S7. The density comparison of transposable elements between M. ruthenica and M. truncatula. Figure S8. Genome synteny between M. ruthenica and M. truncatula. Figure S9. Synteny analysis between genomes of M. ruthenica and C. arietinum. Figure S10. Synteny analysis between genomes of M. ruthenica and T. pretense. Figure S11. Synteny between M. ruthenica and M. sativa in the level of scaffolds. Figure S12. Distribution of FHY3/FAR1 genes and synteny analysis within M. ruthenica genome. Figure S13. The survival rate comparison of other legume forages and M. ruthenica under drought stress. Figure S14. Principal component analysis plot of genetic variation. Figure S15. The genetic structure of populations. Figure S16. Selective sweep analysis using Fst & θπ. Figure S17. Venn diagram of drought-responsive genes from two M. ruthenica accessions collected from sites of Zhenglanqi and Xinghe. Figure S18. The predicted interaction of proteins encoded by drought-responsive genes in M. ruthenica. Figure S19. The number of genes present in the duplicated paralogous blocks of M. ruthenica and M. sativa. Figure S20. The expression of MrABI5, MrMIPS1, MrDREBs, MrPODs and MrP5CSs in two accessions under drought stress. [file 12915_2021_1033_MOESM1_ESM.pdf]

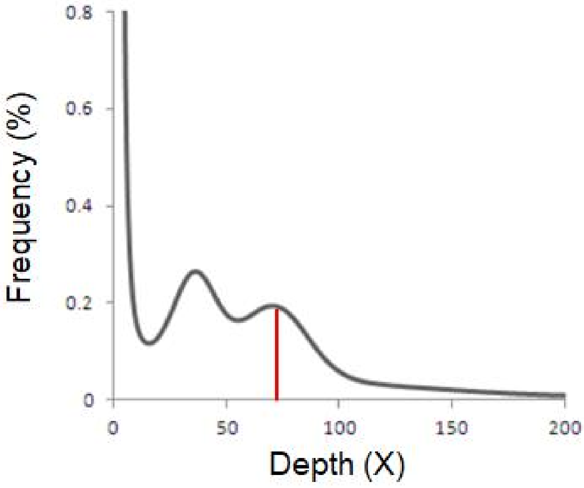

Figure S1 Distribution of 17-mer frequency of *M. ruthenica* genome.

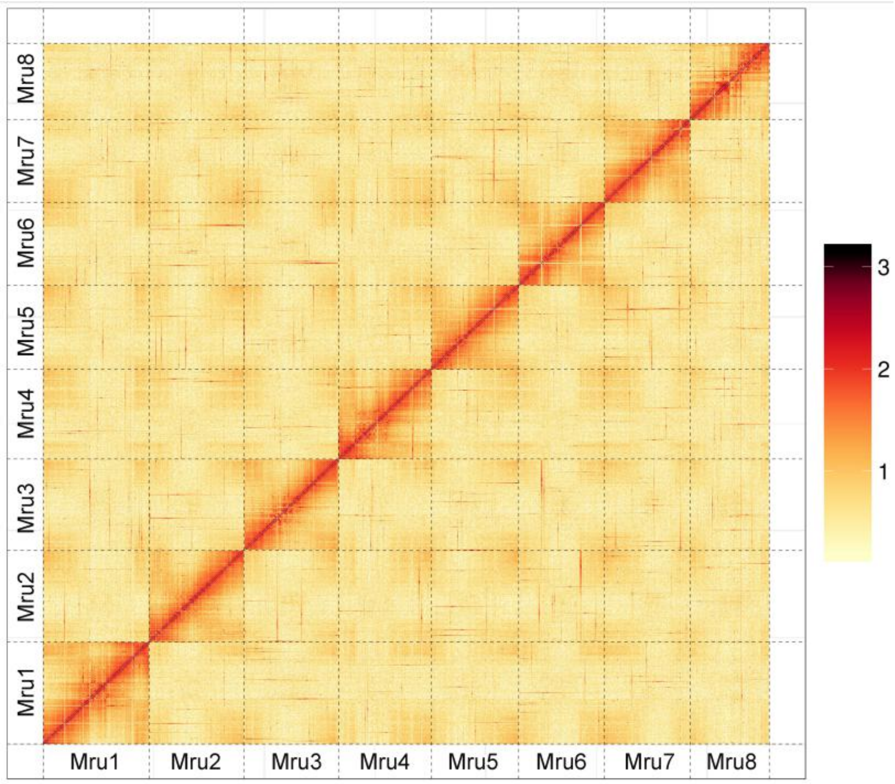

Figure S2 Hi-C interactome within and among chromosomes (Mru1-Mru8).

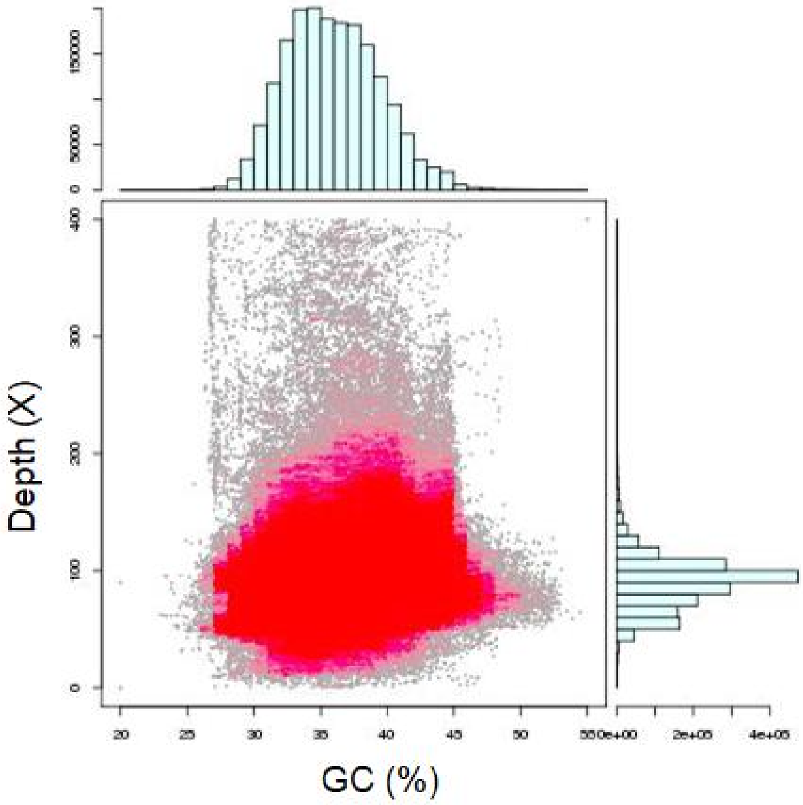

Figure S3 Distribution of GC content and depth.

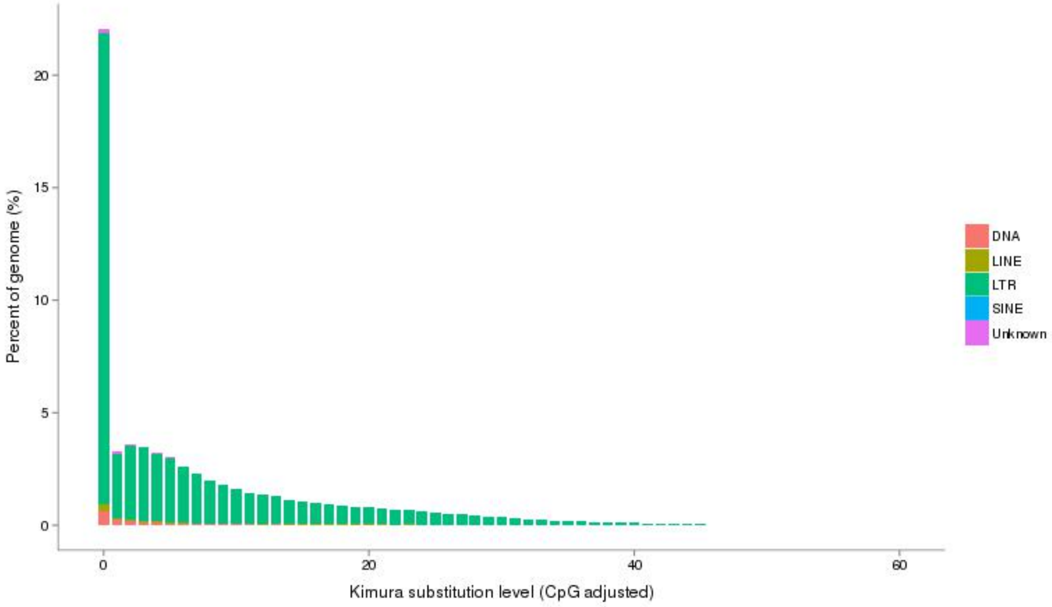

Figure S4 Divergence distribution of classified families of transposable elements. Red indicated DNA transposons (DNA), dark yellow indicated long interspersed nuclear element (LINE), green indicated long terminal repeat (LTR), light blue indicated short interspersed nuclear element (SINE), and purple indicated unknown transposable elements.

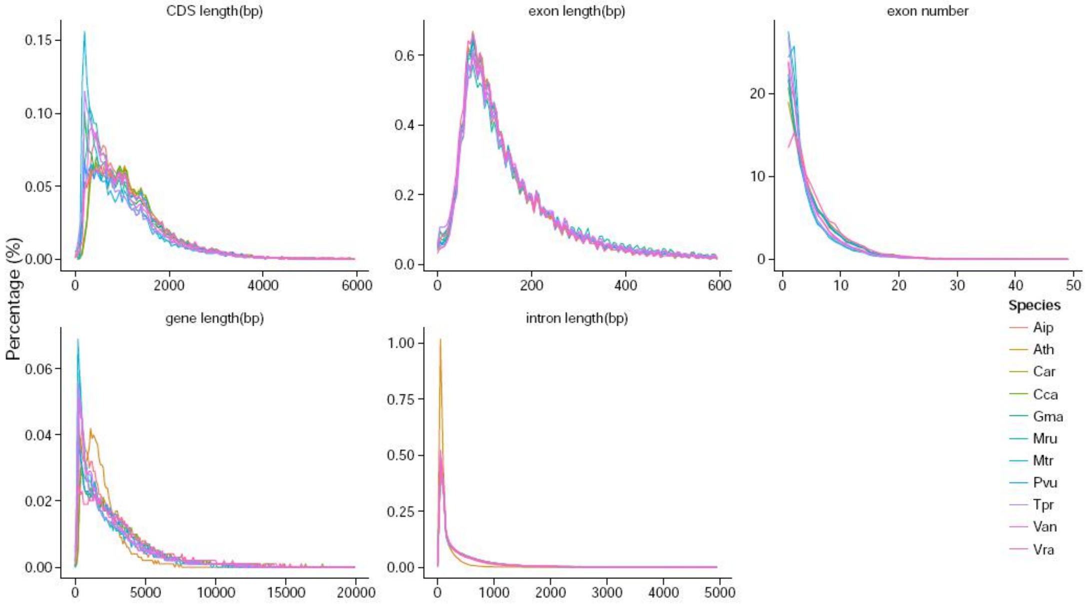

Figure S5 Comparison of gene parameters among *Medicago ruthenica* genome and other genomes. Aip: *Arachis ipaensis*; Ath: *Arabidopsis thaliana*; Car: *Cicer arietinum*; Cca: *Cajanus cajan*; Gma: *Glycine max*; Mru: *Medicago ruthenica*; Mtr: *Medicago truncatula*; Pvu: *Phaseolus vulgaris*; Tpr: *Trifolium pratense*; Van: *Vigna angularis*; Vra: *Vigna radiata*.

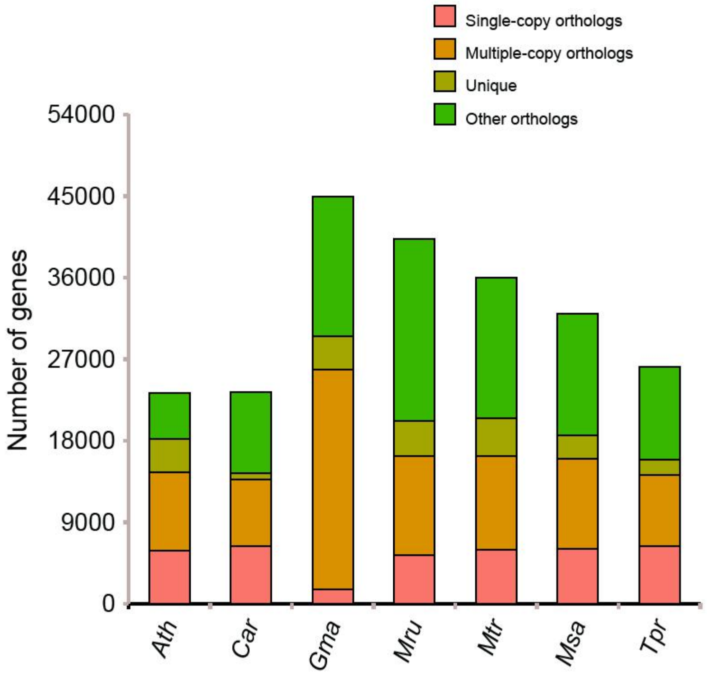

Figure S6 The distribution of genes in different species. Pink indicated single-copy orthologs, yellow indicated multiple-copy orthologs, dark yellow indicated unique genes, and green indicated other orthologs. *Ath*: *Arabidopsis thaliana*; *Car*: *Cicer arietinum*; *Gma*: *Glycine max*; *Mru*: *Medicago ruthenica*; *Mtr*: *Medicago truncatula*; *Msa*: *Medicago sativa*; *Tpr*: *Trifolium pratense*.

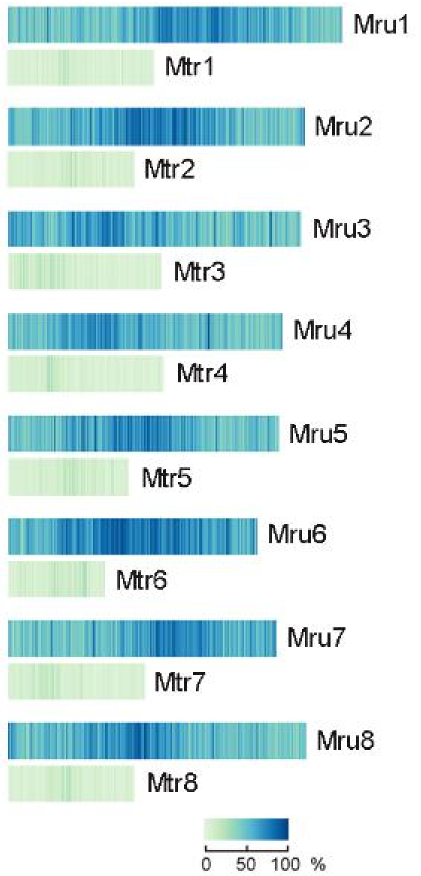

Figure S7 The density comparison of transposable elements between *M. ruthenica* and *M. truncatula*. Mru: *M. ruthenica*; Mtr: *M. truncatula*.

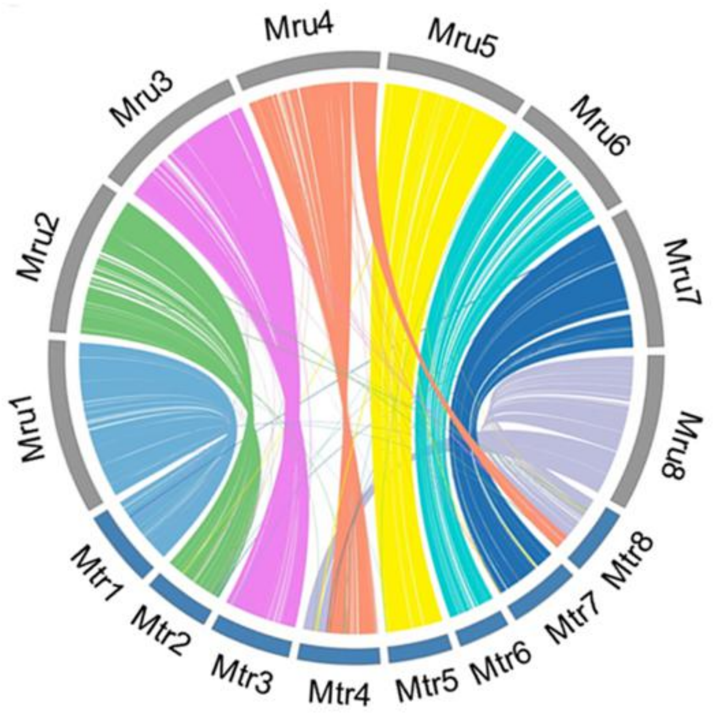

Figure S8 Genome synteny between *M. ruthenica* and *M. truncatula*. Mru: *M. ruthenica*; Mtr: *M. truncatula*.

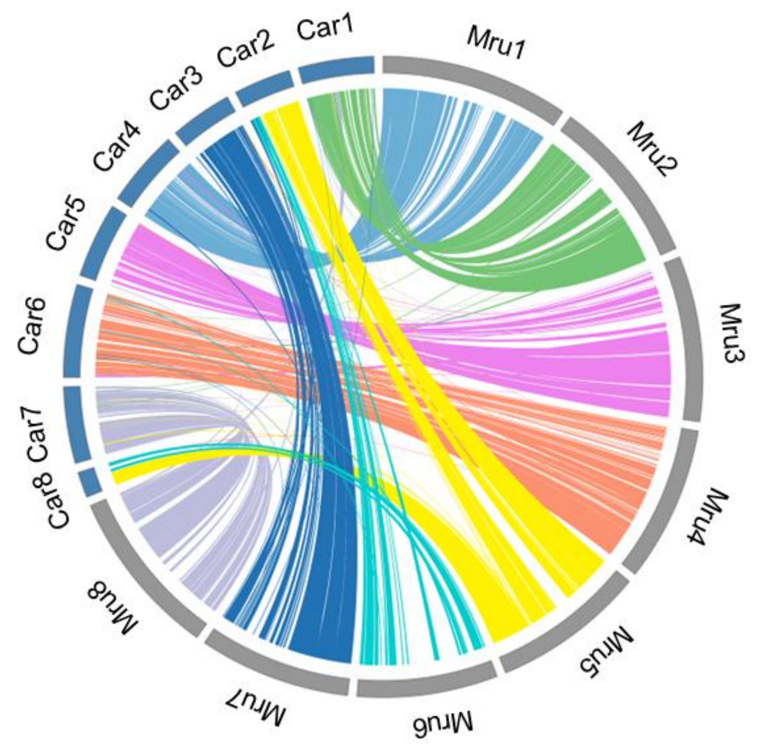

Figure S9 Synteny analysis between genomes of *M. ruthenica* and *C. arietinum.* Mru: *M. ruthenica*; Car: *C. arietinum*.

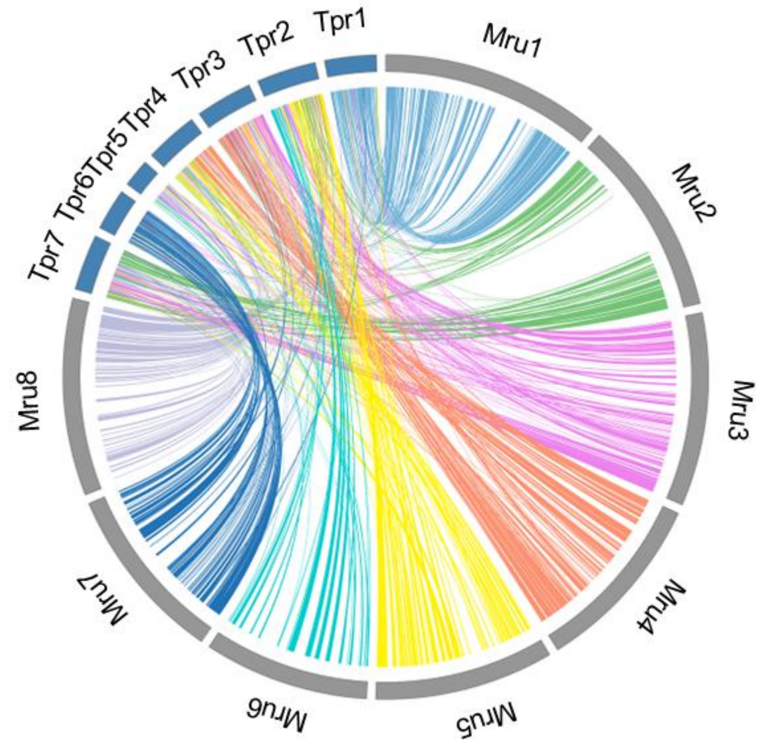

Figure S10 Synteny analysis between genomes of *M. ruthenica* and *T. pretense*. Mru: *M. ruthenica*; Tpr: *T. pretense*.

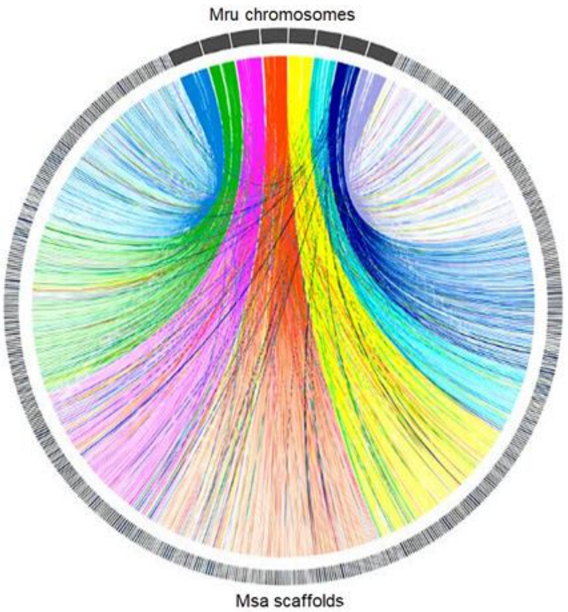

Figure S11 Synteny between *M. ruthenica* and *M. sativa* in the level of scaffolds. Mru: *M. ruthenica*; Msa: *M. sativa*.

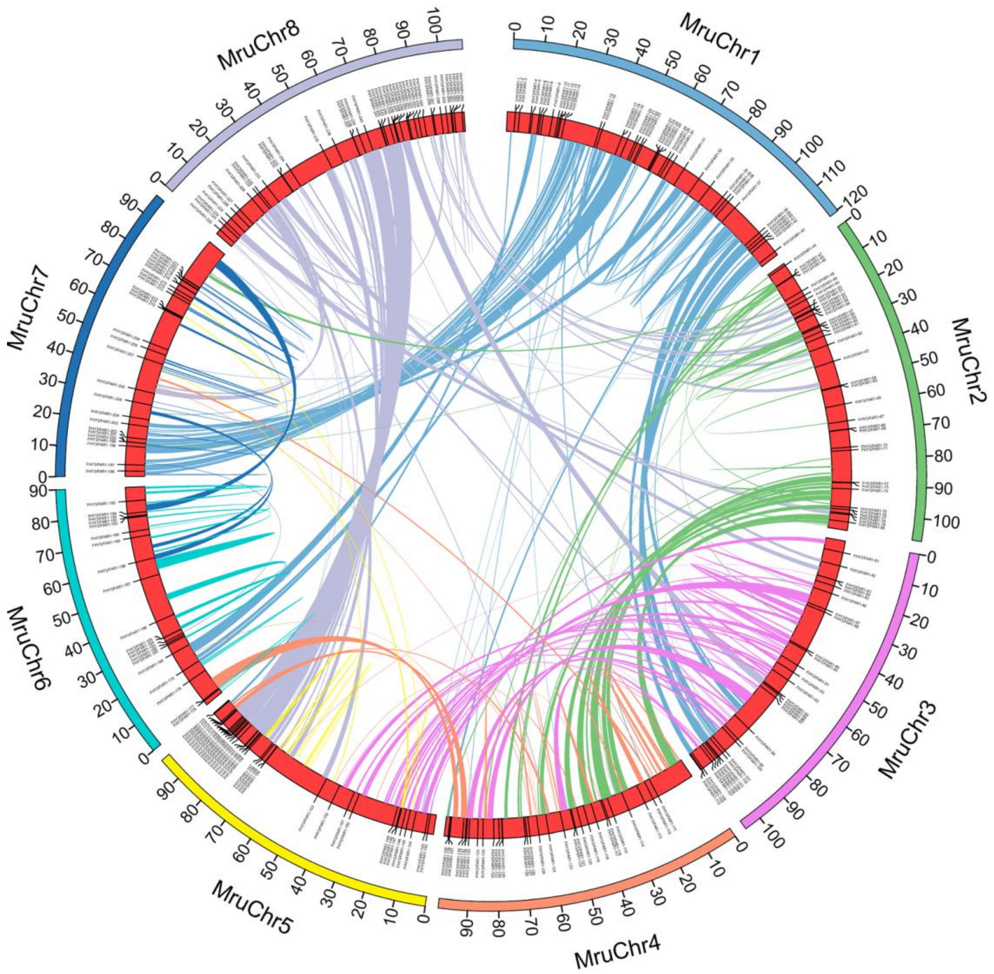

Figure S12 Distribution of *FHY3*/*FAR1* genes and synteny analysis within *M. ruthenica* genome.

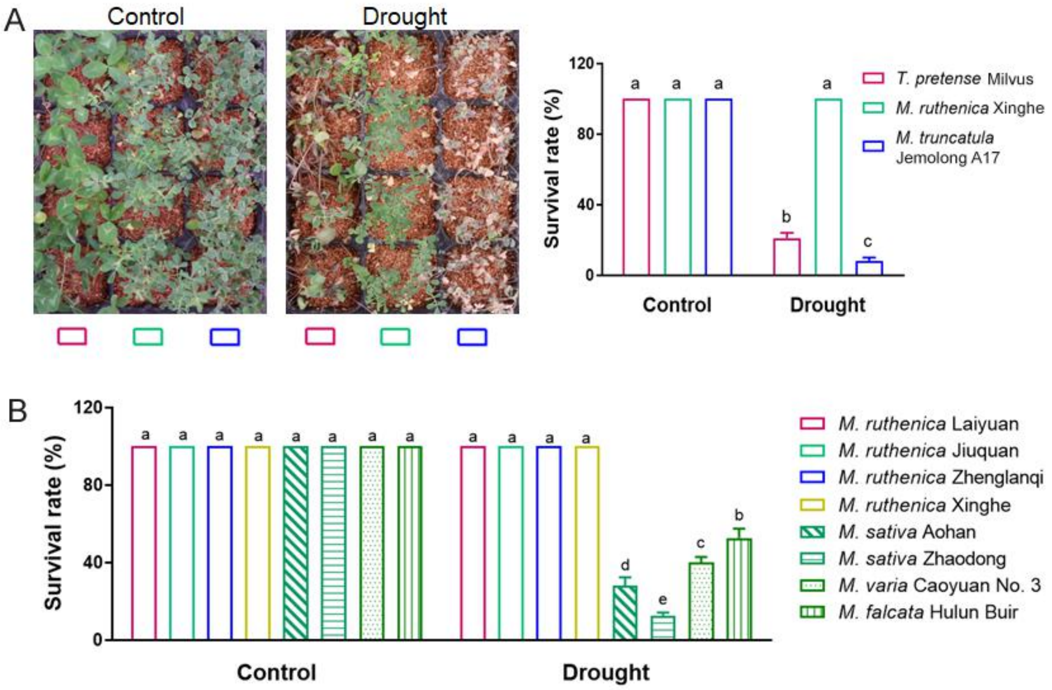

Figure S13 The survival rate comparison of other legume forages and *M. ruthenica* under drought stress. Data are means ± SE (n=4). Different letters mean significant differences among treatments at *P*<0.05. The survival rate at the tenth day of drought was counted.

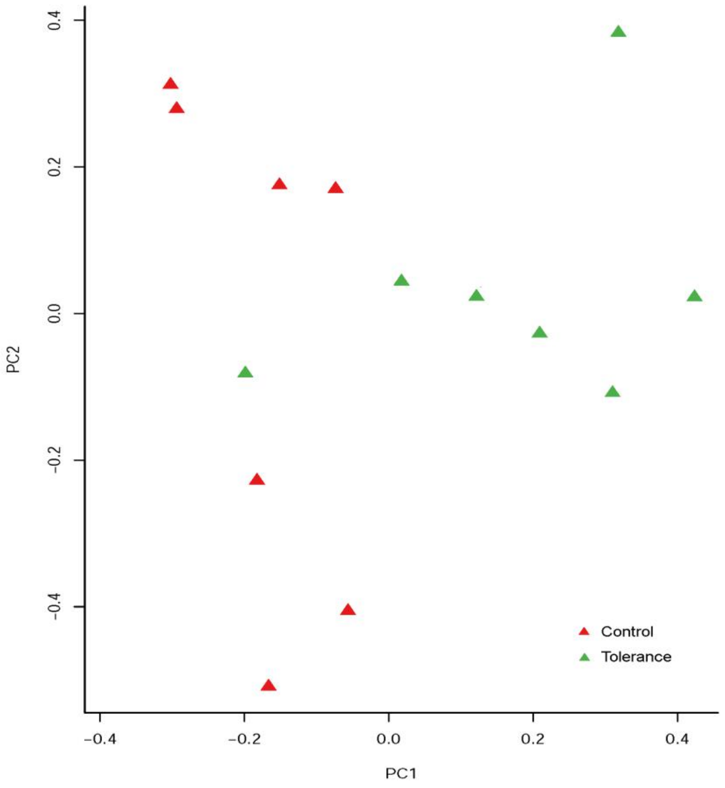

Figure S14 Principal component analysis plot of genetic variation.

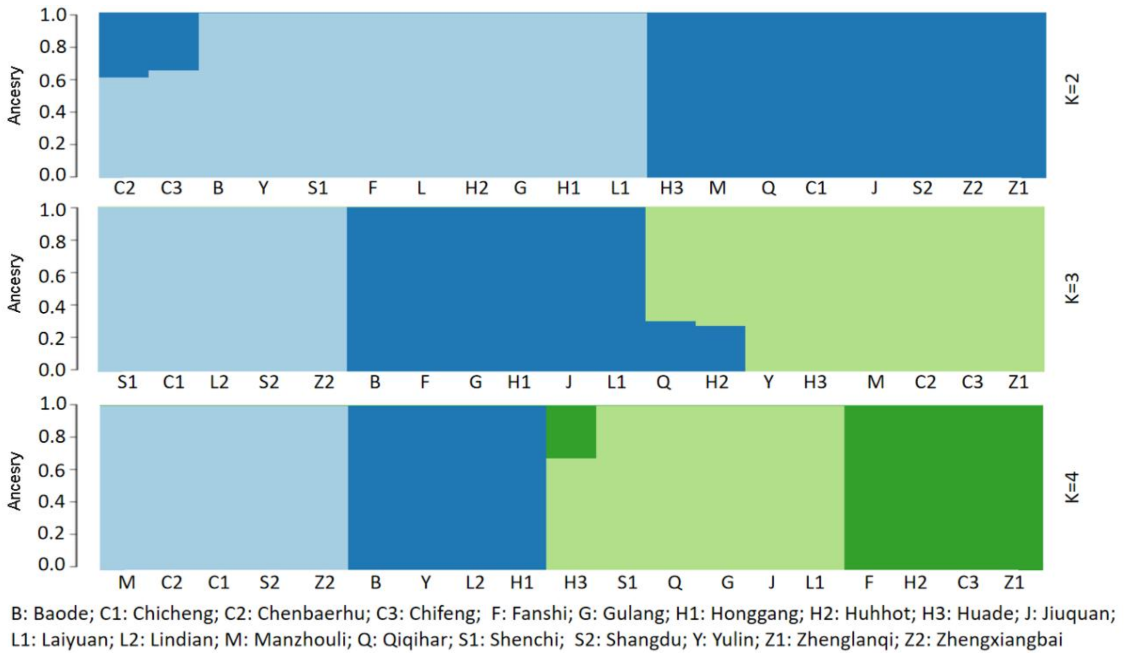

Figure S15 The genetic structure of populations. The length of each colored segment represents the proportion of the individual's genome from K=2~4 ancestral genetic groups.

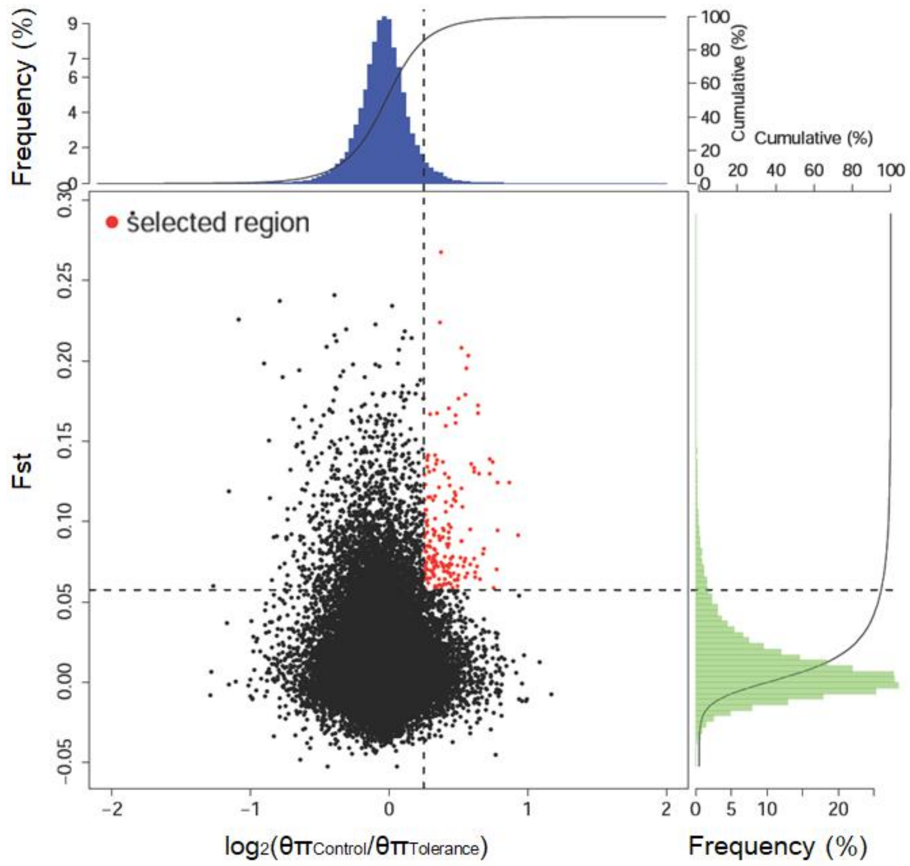

Figure S16 Selective sweep analysis using Fst & θπ. Red points indicate the selected region under drought stress with top 5% Fst and $\log_2(\theta\pi_{Control}/\theta\pi_{Tolerance})$.

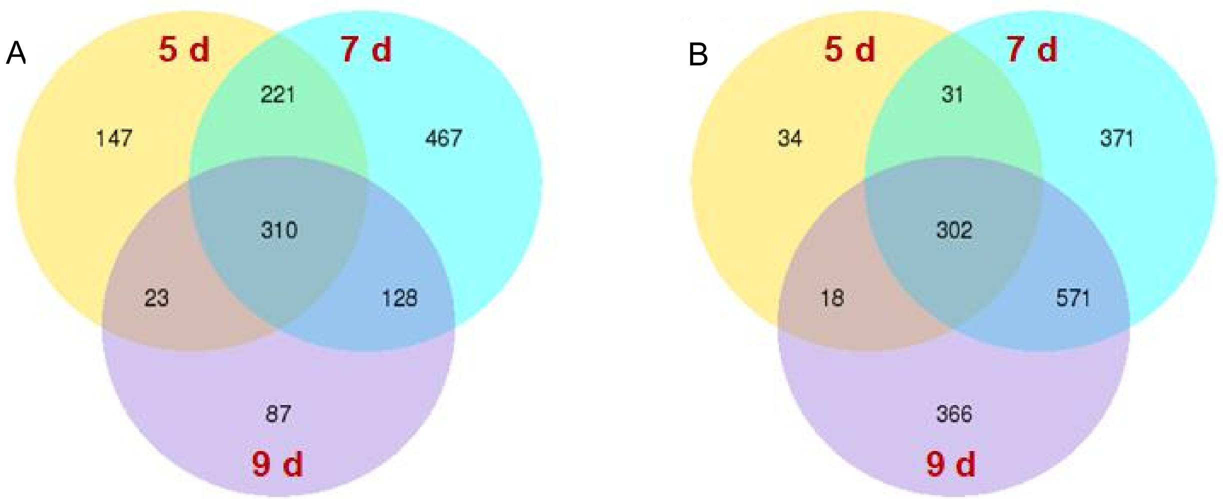

Figure S17 Venn diagram of drought-responsive genes from two *M. ruthenica* accessions collected from sites of Zhenglanqi (A) and Xinghe (B).

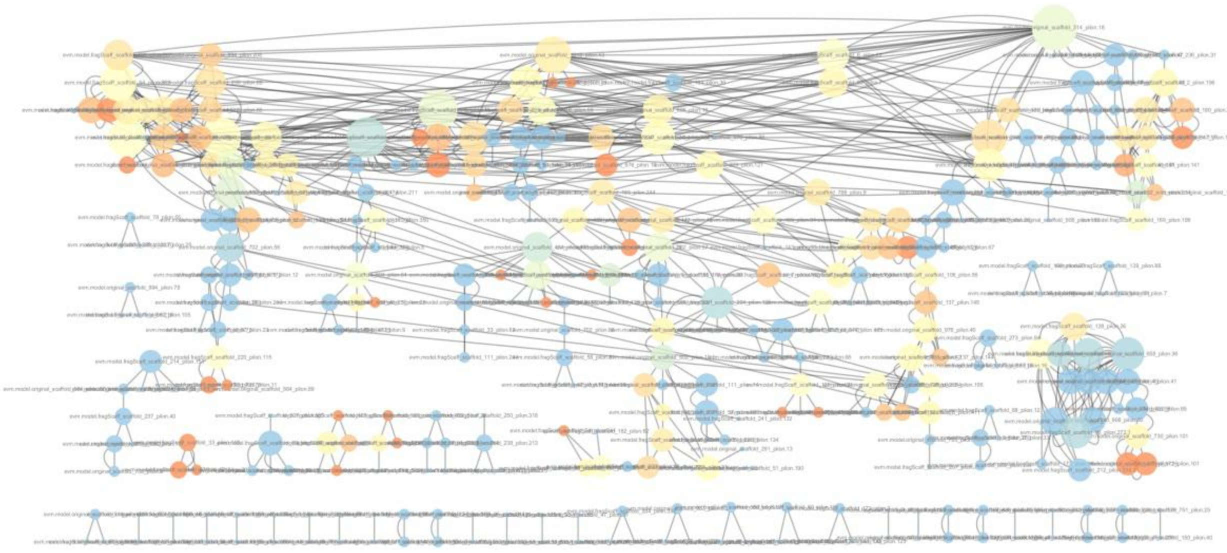

Figure S18 The predicted interaction of proteins encoded by drought-responsive genes in *M. ruthenica*. The network was constructed using STRING.

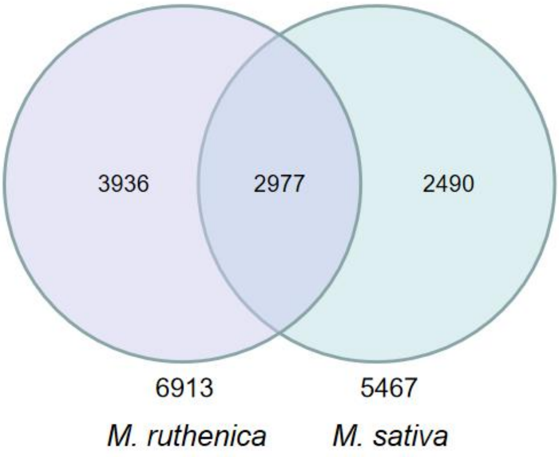

Figure S19 The number of genes present in the duplicated paralogous blocks of *M. ruthenica* and *M. sativa*. The overlapping region represents the genes which are orthologous genes between *M. ruthenica* and *M. sativa*.

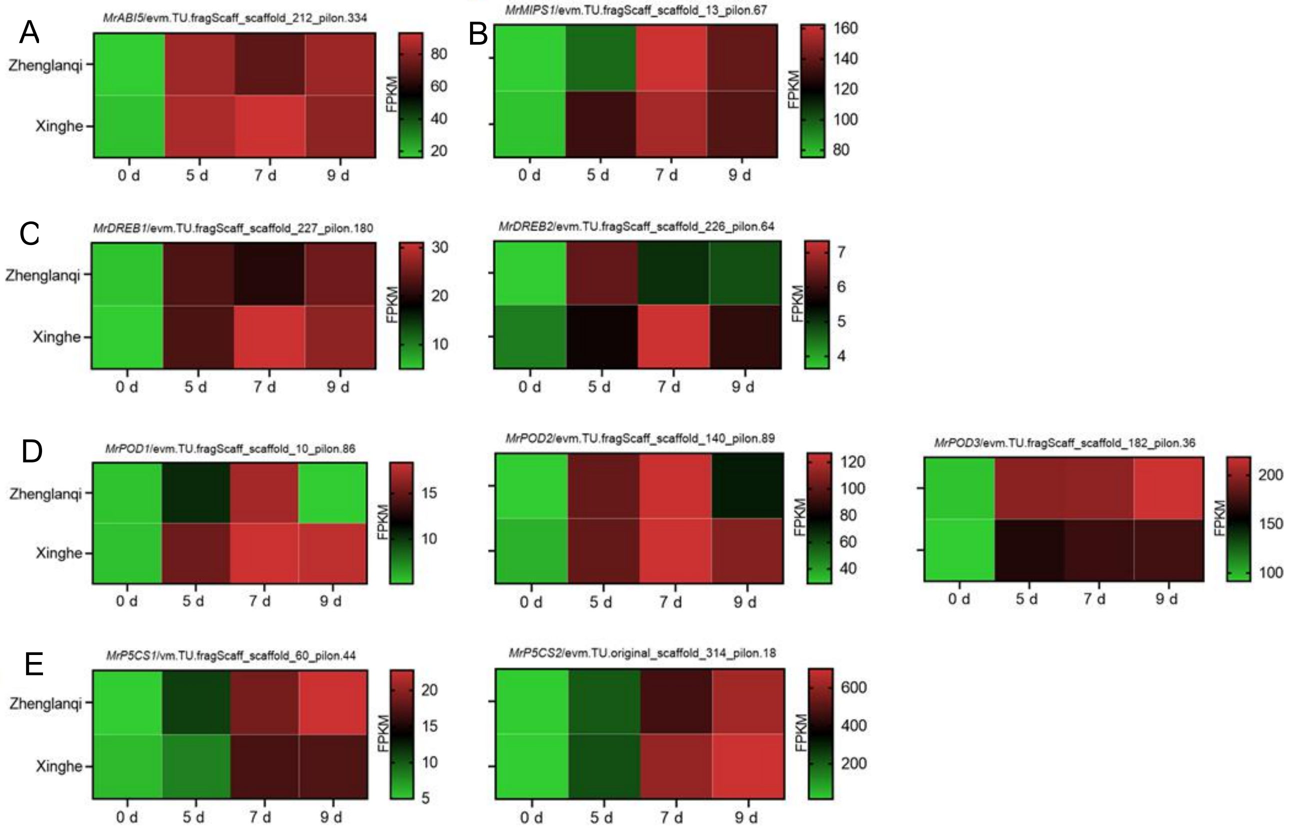

Figure S20 The expression of *MrABI5* (A), *MrMIPS1* (B), *MrDREBs* (C), *MrPODs* (D) and *MrP5CSs* (E) in two accessions under drought stress.
